# A Comprehensive Analysis of the Downregulation of *miRNA-1827* and Its Prognostic Significance by Targeting *SPTBN2* and *BCL2L1* in Ovarian Cancer

**DOI:** 10.3389/fmolb.2021.687576

**Published:** 2021-06-11

**Authors:** Penghui Feng, Zhitong Ge, Zaixin Guo, Lin Lin, Qi Yu

**Affiliations:** ^1^ Department of Obstetrics and Gynecology, Peking Union Medical College Hospital, Chinese Academy of Medical Sciences and Peking Union Medical College, Beijing, China; ^2^ Department of Ultrasound, Peking Union Medical College Hospital, Chinese Academy of Medical Sciences and Peking Union Medical College, Beijing, China; ^3^ Department of Obstetrics and Gynecology, The Maternal and Child Health Hospital of Guangxi Zhuang Autonomous Region, Beijing, China

**Keywords:** miRNA-1827, microRNA-1827, SPTBN2, BCL2L1, Ovarian Cancer

## Abstract

**Background:** Previous studies demonstrated that *miRNA-1827* could repress various cancers on proliferation, angiogenesis, and metastasis. However, little attention has been paid to its role in ovarian cancer as a novel biomarker or intervention target, especially its clinical significance and underlying regulatory network.

**Methods:** A meta-analysis of six microarrays was adopted here to determine the expression trend of *miRNA-1827*, and was further validated by gene expression profile data and cellular experiments. We explored the functional annotations through enrichment analysis for the differentially expressed genes targeted by *miRNA-1827*. Subsequently, we identified two hub genes, *SPTBN2* and *BCL2L1*, based on interaction analysis using two online archive tools, miRWALK (it consolidates the resources of 12 miRNA-focused servers) and Gene Expression Profiling Interactive Analysis (GEPIA). Finally, we validated their characteristics and clinical significance in ovarian cancer.

**Results:** The comprehensive meta-analysis revealed that *miRNA-1827* was markedly downregulated in clinical and cellular specimens. Transfection of the *miRNA-1827* mimic could significantly inhibit cellular proliferation. Concerning its target genes, they were involved in diverse biological processes related to tumorigenesis, such as cell proliferation, migration, and the apoptosis signaling pathway. Moreover, interaction analysis proved that two hub genes, *SPTBN2* and *BCL2L1*, were highly associated with poor prognosis in ovarian cancer.

**Conclusion:** These integrated bioinformatic analyses indicated that *miRNA-1827* was dramatically downregulated in ovarian cancer as a tumor suppressor. The upregulation of its downstream modulators, *SPTBN2* and *BCL2L1*, was associated with an unfavorable prognosis. Thus, the present study has identified *miRNA-1827* as a potential intervention target for ovarian cancer based on our bioinformatic analysis processes.

## Introduction

Ovarian cancer (OV), as one of the three major gynecological malignancies, poses a severe threat to women’s life quality and reproductive health (Siegel et al., 2019). Annually, there are ∼300,000 new cases worldwide identified as OV, with the highest mortality rate among all gynecological malignancies (Bray et al., 2018). OV is classified into various histopathological types, and epithelial cancer is the most common type. Currently, nearly 65–75% of patients are already in the middle or advanced stages when they are first confirmed because the tumor lesions are always located deep in the pelvic cavity, and there are no practical approaches for screening and diagnosis for early onset. Although ∼75% of patients can benefit from the initial treatment with impermanent remission, almost all terminal cases will develop into the recurrent and multidrug-resistant status, and the 5-year survival rate of OV patients is merely 30–50% (Salani et al., 2017; Allemani et al., 2018).

Recently, the development of bioinformatics has promoted the exploration of various kinds of cancers by integrating data resources and clinical information, which assists experimental biologists in carrying forward these findings into clinical validation and application (Fu et al., 2020; Udhaya Kumar et al., 2020a). Previous studies have determined the correlation between the aberrant expression of specific genes and oncogenesis and progression of OV, and unveiled the predictive potential of these biomarkers for prognosis (Zheng et al., 2019; Yang et al., 2020). As reported, a study has analyzed energy metabolism–associated characteristics to evaluate the prognosis of patients with OV by nonnegative matrix factorization clustering analysis; it finally established an eight-gene signature associated with metabolic genes (Wang and Li, 2020). Another study has also identified four core genes associated with the prognosis of OV using the Analyze Networks algorithm (Kumar et al., 2019). At the same time, mounting evidence has revealed the latent significance of miRNAs as new diagnostic and prognostic markers based on bioinformatic analyses for the individualized treatment of OV.

miRNAs are single-stranded, noncoding, small RNAs of approximately 22 nucleotides in length, generated from endogenous heparin loop miRNA precursors, which are enrolled in the regulation of gene expression at the transcriptional or posttranscriptional level (Kim, 2005; Graves and Zeng, 2012). Additionally, existing research recognizes the pivotal role of miRNAs in many biological behaviors of cancers, as penetratingly analyzed in the previous studies, including angiogenesis, tissue invasion or metastasis, deregulating cell energetics, and avoiding immune destruction (Bueno et al., 2008; Nicoloso et al., 2009; Hanahan and Weinberg, 2011; Fei et al., 2012; Wei et al., 2013). In particular, the aberrant expression of some ovary-specific miRNAs can be a crucial putative biomarker or indicator in the diagnosis, individualized treatment, and prognosis of OV (Pal et al., 2015). For example, *miR-182* was confirmed to be upregulated in OV cases using a public dataset from the Gene Expression Omnibus (GEO) database, and two hub genes, *MCM3* and *GINS2*, were indicated to be associated with the worse overall survival of patients (Li and Li, 2019). Based on these predictive hallmarks, miRNAs involved in tumorigenesis and progression have aroused wide attention.

*microRNA-1827* or *miRNA-1827*, as a novel miRNA, acts with multiple biological functions in various types of cancers. *miRNA-1827* was initially identified in HeLa cells by miRDeep sequencing (Friedlander et al., 2008). A recent study demonstrated that it could tightly regulate the expression level and function of *TP53* to suppress the oncogenesis of human colorectal cancer by targeting *MDM2*, which could bind to and degrade the P53 protein under ubiquitylation (Zhang et al., 2016). Furthermore, similar research in ulcerative colitis patients also indicated that *miRNA-1827* in serum was decreased and was correlated with a higher risk of colorectal cancer (Polytarchou et al., 2015). Besides, *miRNA-1827* was a putative therapeutic target in lung cancer *in vitro* and *in vivo*, and it could modulate the migration, angiogenesis, or invasion directly by targeting the *CRKL* molecule (Ho et al., 2014; Ho et al., 2018). However, as much as it has been reported to be downregulated in most types of cancers as a tumor repressor, very few studies have investigated its role in OV. Therefore, it will be essential to verify its clinical significance as a new intervention target and reveal the underlying mechanisms by screening out the possible targets of *miRNA-1827* for further application.

This study assessed the influences of the differential expression of *miRNA-1827*, with all data collected from six GEO microarrays. To illuminate the potential modulatory mechanisms, the downstream regulators targeted by *miRNA-1827* were predicted and verified by 12 prophetic databases together with the Gene Expression Profiling Interactive Analysis (GEPIA) database (the latter is based on the Cancer Genome Atlas (TCGA) and the Genotype-Tissue Expression (GTEx) data). Subsequently, Gene Oncology (GO) enrichment, Kyoto Encyclopedia of Genes and Genomes (KEGG), and protein–protein interaction (PPI) network analysis were comprehensively performed.

## Materials and Methods

### Gene Expression Omnibus Microarray Acquisition

**FIGURE 1:**
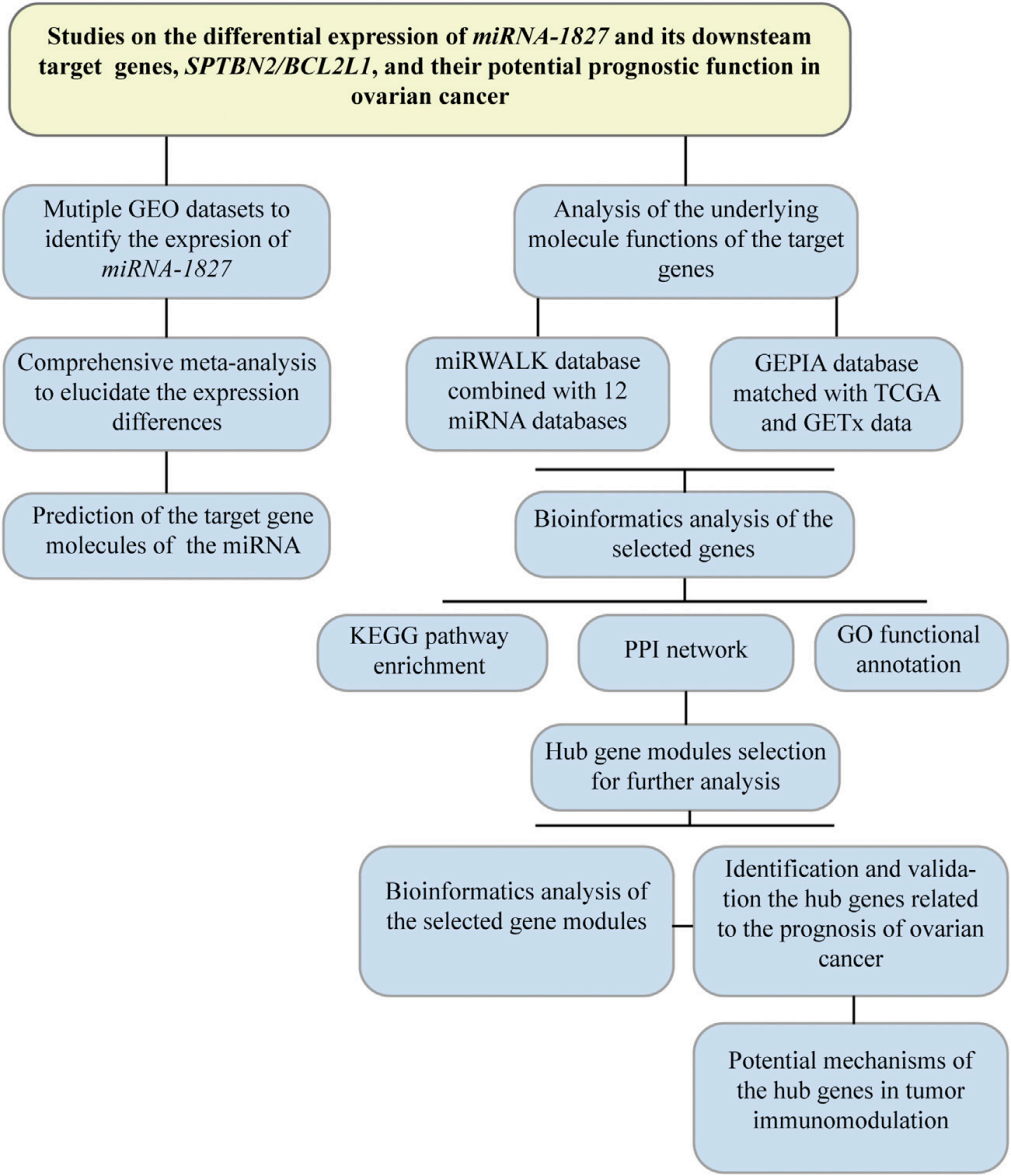
Flow diagram of the research design. This flowchart presented a comprehensive meta-analysis to validate the expression among different microarrays in OV, and bioinformatic analysis was performed to investigate the putative target genes. Hub genes were filtered out for further research on their prognostic significance.

To retrieve the microarray data on *miRNA-1827* in OV, we searched the GEO database (https://www.ncbi.nlm.nih.gov/geo/) with the keywords as listed: ((“ovarian” OR “ovary” OR “ovaries”) AND (“cancer” OR “cancerous” OR “carcinoma” OR “carcinomatous” OR “neoplasm” OR “neoplasms” OR “adenoma” OR “adenomas” OR “adenocarcinoma” OR “adenocarcinomas” OR “malignancy” OR “tumor” OR “tumors”) AND (“micro RNA” OR “microRNA” OR “miRNA” OR “non-coding RNA” OR “ncRNA” OR “small RNA”)). The retrieval expression was filtered by “*Homo sapiens*” of the organism. In addition, the inclusion criteria were set as follows: 1) patients diagnosed with OV and its subtypes were included; 2) the experiments were performed with cancerous samples and normal control (NC), and for at least three, biological duplications were investigated; 3) the bio-specimens were isolated from tissue, serum, or urine; and 4) the expression profiling data of *miRNA-1827* were available. All data were processed according to the flow diagram, as shown in Figure 1.

### Statistical Comparison and Comprehensive Meta-Analysis for Microarrays

The expression profiling results of *miRNA-1827* derived from the queried microarrays were aggregated to calculate the number, mean value (M), and standard deviation (SD) for comparisons between the two groups by the Student’s t-test, and the expressional results were displayed using the ggpubr package. Then, a comprehensive meta-analysis by the meta package was performed to evaluate the data from all selected microarrays, for which the expression of *miRNA-1827* between groups was illustrated by the forest plot that displayed the standardized mean difference (SMD) and the 95% confidential interval. It should be mentioned that the DerSimonian–Laird estimator for τ^2^ and I^2^ statistics was applied to determine whether to adopt a random-effects model or fixed-effects model for the pooled estimate. The sensitivity analysis was conducted to assess the heterogeneity among different studies, and if it did exist, the subgroup analysis was carried out to ascertain the reason for the heterogeneity. To measure publication bias, Begg’s test and funnel plots were used.

### Roles of *miRNA-1827* in the Proliferation of Ovarian Cancer Cells

To verify the expression level of *miRNA-1827* in OV cells, five types of OV cell lines (OV1063, Caov3, SKOV3, OVCAR3, and A2780 cells) and two kinds of human normal ovarian epithelial cells (IOSE80 and HOSEpiC) were used for further experiments. As directed, these cells were cultured in the recommended medium in a humidified atmosphere supplemented with 5% CO_2_. Cells were passaged by trypsin digestion at 80% confluence. To quantify the content of *miRNA-1827*, the total miRNAs were extracted by an miRNA Isolation Kit (Vazyme, China). The purified miRNAs were then pretreated with DNase I (5 U/μl) solution. The miRNA first-strand synthesis kit (Clontech, United States) was applied to convert miRNAs into cDNA. After that, a qRT-PCR reaction was carried out using the Mir-X miRNA qRT-PCR TB Green^®^ kit (Clontech, United States). PCR primer sequences (*miRNA-1827* forward primer: GCA​GTG​AGG​CAG​TAG​ATT​G, *miRNA-1827* reverse primer was provided by the kit; *U6* forward primer GGA​ACG​ATA​CAG​AGA​AGA​TTA​GC, *U6* reverse primer: TGG​AAC​GCT​TCA​CGA​ATT​TGC​G) were designed using miRprimer2 software (Busk, 2014). Cycling conditions for the PCR machine were set as follows: pre-denaturation at 95°C for 30 s, following amplification condition at 95°C for 5 s, and 60°C for 34 s for 40 cycles. Gene amplification levels were quantified by the delta–delta CT method and standardized to the reference gene.

Based on the quantification results, A2780 and OV1063 cells were seeded onto 25 cm^2^ culture flasks and harvested after rinsing with PBS. Cells were then cultured in 96-well flat plates with 100 μl in each well. In order to determine the effects of *miRNA-1827* on the proliferation of OV cells, the riboFECT CP transfection kit (RiboBio, China) was used to transfect the *miRNA-1827* mimic or inhibitor into A2780 and OV1063 cells, respectively. In detail, a 50 nM mimic (sense strand: UGA​GGC​AGU​AGA​UUG​AAU, antisense strand: ACU​CCG​UCA​UCU​AAC​UUA) or 100 nM inhibitor (sequence: mAmUmUmCmAmAmUmCmUmAmCmUmGmCmCmUmCmAm) was added into the corresponding medium mixed with 6 μl of buffer and 0.6 μl of transfection reagent. Cells were incubated for 48 h. Two hours before the end of the incubation, 10 μl of CCK-8 reagent was added (Vazyme, China) into each well. After that, the optical density was detected at 450 nm using a microplate reader.

### Putative Targets of *miRNA-1827* in Ovarian Cancer

To locate the targets for *miRNA-1827* based on miRNA–gene interactions, data mining was processed using an online archived database, miRWALK (http://zmf.umm.uni-heidelberg.de/apps/zmf/mirwalk2/index.html) (Sticht et al., 2018). Twelve classical miRNA-focused servers, including miRWalk, MicroT4, miRanda, miRBridge, miRDB, miRMap, miRNAMap, PICTAR2, PITA, RNA22, RNAhybrid, and Targetscan, were taken into consideration. Target genes were selected only when they were projected by no less than six of the servers mentioned above. The upregulated genes in OV were hunted by the GEPIA database (http://gepia.cancer-pku.cn/) (Tang et al., 2019) with a fold change value of more than one and an adjusted *p*-value < 0.05. The putative target genes were finally recognized from the overlapped genes among the predictive targets from the miRWALK database and the overexpressed genes were acquired in the GEPIA database.

### Functional Enrichment Analysis for Latent Target Genes

GO term enrichment and KEGG analyses have been widely applied to consolidate biology in compiling a disciplined, structured, and defined glossary for numerous genes. The Database for Annotation, Visualization, and Integrated Discovery (DAVID) (https://david.ncifcrf.gov/) (Dennis et al., 2003) was utilized to associate functional terms with the uploaded genes using the clustering algorithms. The enriched results were classified into the biological process, cellular component, and molecular function terms, as revealed by the bubble plots, and integrated functional pathways, as shown in the chordal graph by ggplot2 and GOplot packages.

### Identification and Characteristics of Prognostic Hub Genes

To identify the hub genes that interacted with *miRNA-1827* and were relevant to the clinical prognosis, the latent target genes described above were explored by the PPI analysis *via* the online database, STRING (https://string-db.org/cgi/input.pl) (von Mering et al., 2003). The outcomes were presented by Cytoscape software (https://cytoscape.org/) (Shannon et al., 2003). On this basis, gene modules or clusters were filtered *via* the MCODE plugin with a K-core value > 2. Then, four clusters were screened out, and 34 genes were identified for subsequent bioinformatic analyses. Among these genes, two hub genes, *SPTBN2* and *BCL2L1*, were matched with prognostic significance and validated by the GEPIA survival and correlation analyses.

### Validation of the Expression Patterns of the Hub Genes

To validate the expression patterns of the hub genes, gene expression profiles of OV samples were downloaded from the project of the International Cancer Genome Consortium (ICGC) database (https://dcc.icgc.org/) (Zhang et al., 2019). Besides, data generated from normal tissues were obtained from the GEPIA database as controls. All the data after normalization were processed to identify the expression difference of *SPTBN2* and *BCL2L1* between groups. In order to better understand these two hub genes, the Human Protein Atlas (HPA) database (http://www.proteinatlas.org/) (Uhlen et al., 2015) was further utilized to exploit the relevant information on the location and protein expression in the cancer specimens compared with controls through the cell, tissue, and pathology atlas modules of this tool for further qualitative analyses.

### The Tumor Immunoregulatory Network Coordinated by the Hub Genes

To determine the potential mechanisms of the hub genes in the immunoregulatory system in OV, we explored the Tumor Immune Estimation Resource (TIMER) online tool (https://cistrome.shinyapps.io/timer/) (Li et al., 2017). We investigated the correlation between the hub gene expression and abundance of immune infiltration, and the association between clinical outcome and abundance of immune infiltration. The estimate algorithm was applied to determine the immune–stromal component in the tumor microenvironment (TME) of each sample utilizing the estimate package in R software (https://r-forge.r-project.org/). The limma package was applied to normalize the gene expression profile from the TCGA database to evaluate the proportion of tumor-infiltrating immune cells (TICs), and then a standardized gene expression profile was uploaded to CIBERSORT (Newman et al., 2015). The deconvolution algorithm was introduced here to estimate the TIC abundance. According to the average abundance of CD4^+^ T cells or dendritic cells, Kaplan–Meier analysis by the log-rank test analysis was carried out to assess survival possibility with the differential expression of *SPTBN2* and *BCL2L1*.

## Results

### Confirmation of the Expression of *miRNA-1827* and the Meta-Analysis Results

**FIGURE 2:**
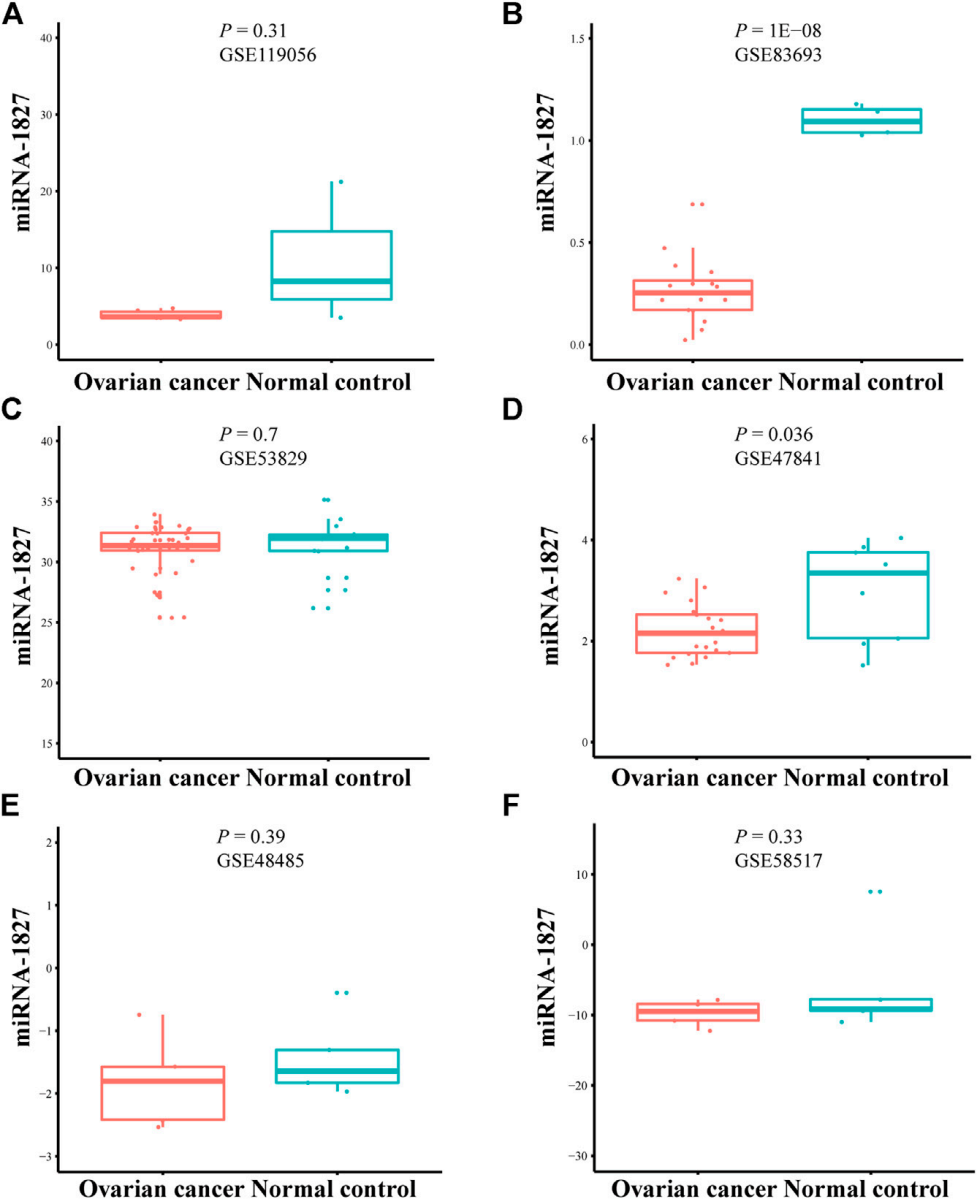
Downregulation of *miRNA-1827* in the selected microarrays from the GEO datasets. **(A)** GSE119056. **(B)** GSE83693. **(C)** GSE53829. **(D)** GSE47841. **(E)** GSE48485. **(F)** GSE58517.

**FIGURE 3:**
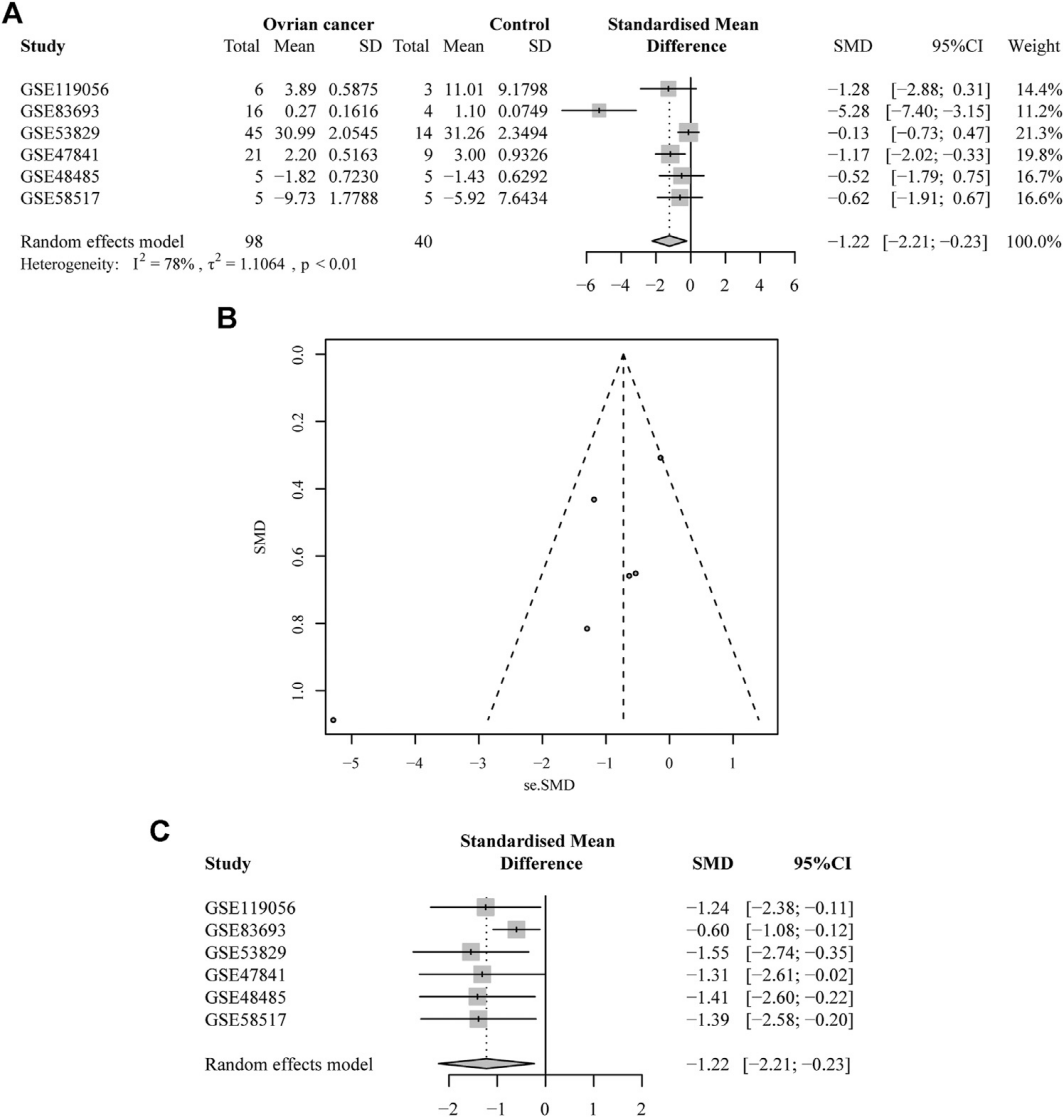
A comprehensive meta-analysis of the expression of *miRNA-1827* from the collected microarrays. **(A)** The forest plot for the pooled SMD of −1.22 (95% CI: −2.21, −0.23) with a degree of heterogeneity (I^2^ = 78%, *p* < 0.01). **(B)** The funnel plot for assessing the publication bias of the GEO datasets by Begg’s test with *p* = 0.09. **(C)** The sensitivity analysis for the GEO microarray results.

There were six microarrays included in this study from the GEO database, as depicted in Table 1. In total, four datasets were collected from tissue samples (GSE119056, GSE83693, GSE53829, and GSE47841), and the rest were from either serum (GSE48485) or urine specimens (GSE58517). Concerning the expression data from those datasets of *miRNA-1827*, this miRNA was downregulated in the OV group compared with the NC group in all enrolled datasets, and two of them were expressed with significance, including GSE83693 (*p* = 1E-08) and GSE47841 (*p* = 0.036), as shown in Figure 2. On this basis, a comprehensive meta-analysis was performed to precisely quantify the expression of *miRNA-1827*. The calculation results are displayed in Figure 3A, for which the random-effects model was applied considering the existing heterogeneity (I^2^ = 78%, τ^2^ = 1.1064, *p* < 0.01). Results also indicated that *miRNA-1827* was remarkably downregulated in the OV group (SMD = −1.2239, 95% CI [−2.2145; −0.2333], Z = −2.42, *p* = 0.0155). To clarify the source of heterogeneity, publication bias was evaluated, as shown in Figure 3B. The symmetric funnel plot signified there was no publication bias (z = −1.6908, *p*-value = 0.09087). Later, a sensitivity test was generated to assess the significant heterogeneity, and no items were found to have a particular effect on the results (Figure 3C).

**TABLE 1:** Gene Expression Omnibus datasets involved.

Accession	GPL	Year	Ovarian cancer			Normal control			Source
			*n*	M	SD	*n*	M	SD	
GSE119056	GPL21572	2019	6	3.89	0.59	3	11.01	9.18	Tissue
GSE83693	GPL22079	2017	16	0.27	0.16	4	1.10	0.07	Tissue
GSE53829	GPL18138	2014	45	30.99	2.05	14	31.26	2.35	Tissue
GSE47841	GPL14613	2014	21	2.20	0.52	9	3.00	0.93	Tissue
GSE48485	GPL14943	2014	5	−1.82	0.72	5	−1.43	0.63	Serum
GSE58517	GPL18402	2015	5	−9.73	1.78	5	−5.92	7.64	Urine

M, mean; SD, standard deviation.

**FIGURE 4:**
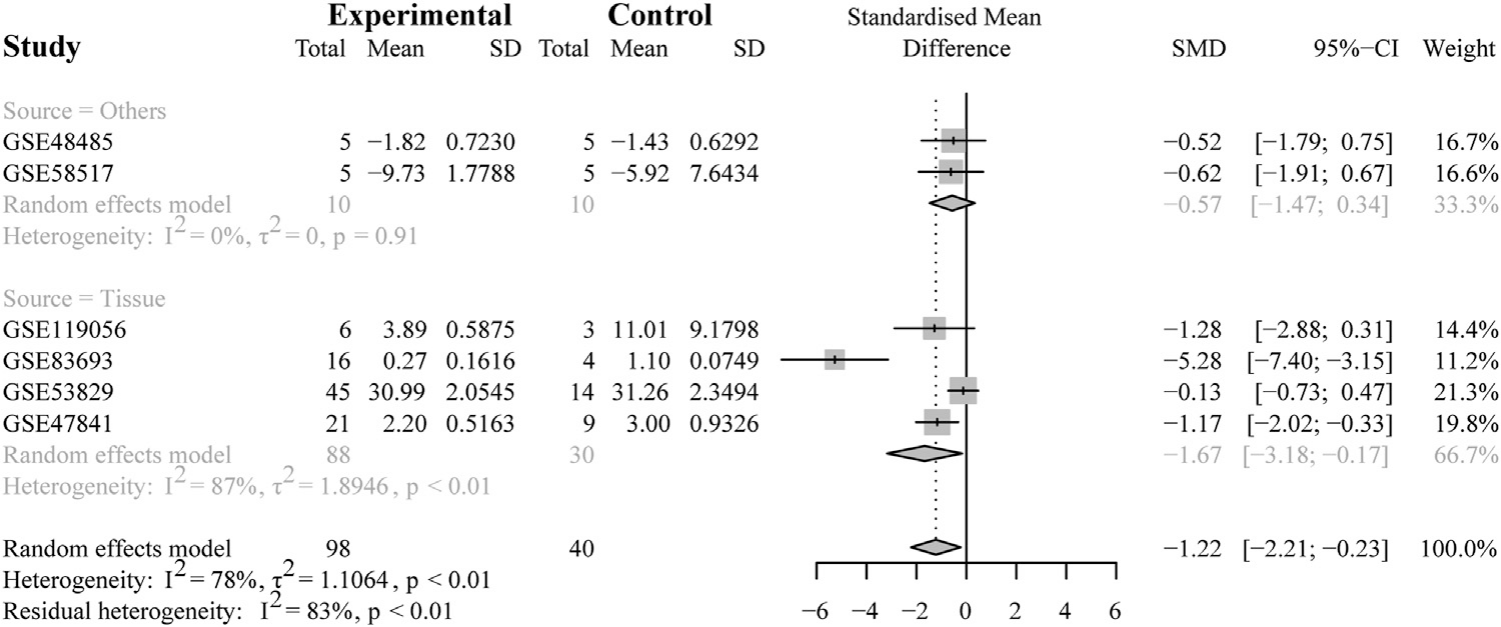
Results of the subgroup analysis. The subgroup analysis based on sample sources (non-tissue or tissue). The result indicated that the tissue subgroup had obvious heterogeneity (I^2^ = 87%, *p* < 0.01).

To further validate the heterogeneity, subgroup analysis was conducted according to the source types, divided into two groups (the non-tissue group or tissue group), as depicted in Figure 4. The results demonstrated that no heterogeneity was observed in the non-tissue group (SMD = −0.57, 95% CI [−1.47; −0.34], Q = 0.01, τ^2^ = 0, I^2^ = 0.0%). Conversely, the tissue group was found to account for the heterogeneity (SMD = −1.67, 95% CI [−3.18, −0.17], Q = 22.90, τ^2^ = 1.8946, I^2^ = 87%). In this group, the study (GSE53829) might be the main cause with the largest number of samples included in the microarrays (45 from the OV samples and 14 from the NC samples).

Subsequently, we explored the expression of *miRNA-1827* in OV cells, and our results indicated that these cell lines exhibited consistent trends of declined expression in these five kinds of OV cells compared with the normal ovarian epithelial cells, as shown in Supplementary Figure S1A. Notably, there existed significant differences of *miRNA-1827* in A2780 cells. Similarly, the expression level of *miRNA-1827* in OVCAR3 cells differed from that of *miRNA-1827* in HOSEpiC cells. According to these results, two cells, A2780 and OV1063, were chosen to be transfected with the mimic or inhibitor of *miRNA-1827*, respectively. A CCK8 assay was performed after transfection to determine the cell viability, and we found that the proliferation of A2780 cells was dramatically inhibited after being treated with the *miRNA-1827* mimic (*p* < 0.01). However, the competitive inhibition of *miRNA-1827* seemed not to make a meaningful difference, which might be reasonably explained by its relatively low content in OV cells (Supplementary Figure S1B).

### Functional Enrichment Results of the Target Genes and Their Core Modules

**FIGURE 5:**
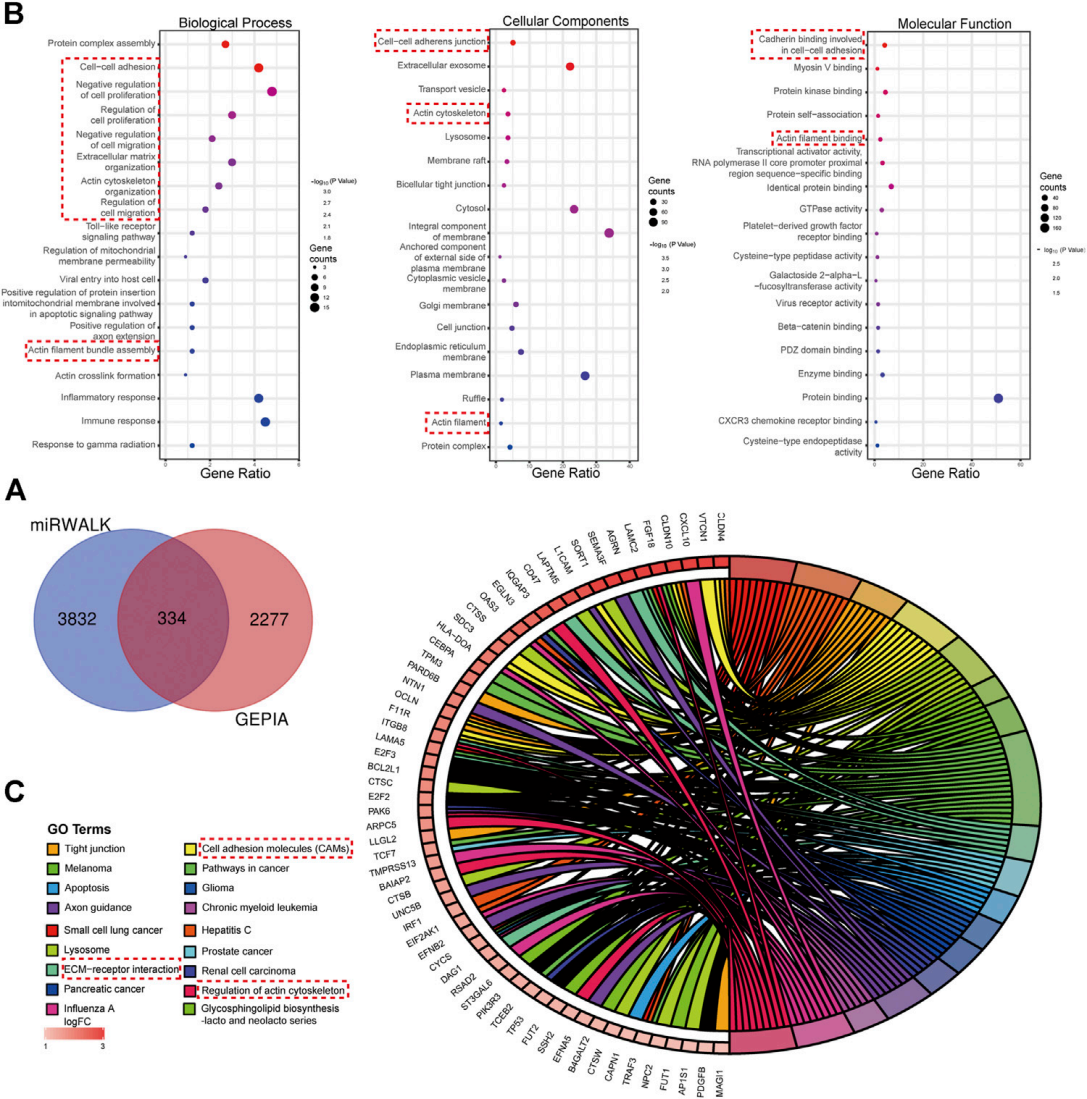
Target genes interacted with *miRNA-1827* and the functional enrichment analysis for these genes. **(A)** The Venn diagram for the overlapped targets predicted by different databases, with 4,166 genes from the miRWALK tool based on 12 servers and 2,611 highly expressed genes from the GEPIA tool based on the TCGA and GTEx databases (adjusted *p* < 0.05). **(B)** GO enrichment analysis processed by the DAVID online tool including the biological process (left panel), cellular component (middle panel), and molecular function (right panel), as shown in the bubble plots. The size of the circles referred to the gene counts, and the X-axis pointed to the gene ratio. **(C)** KEGG pathway enrichment. The chordal diagram displayed the pathway terms enriched in the KEGG database. The input genes derived from the highly expressed predicted targets.

**FIGURE 6:**
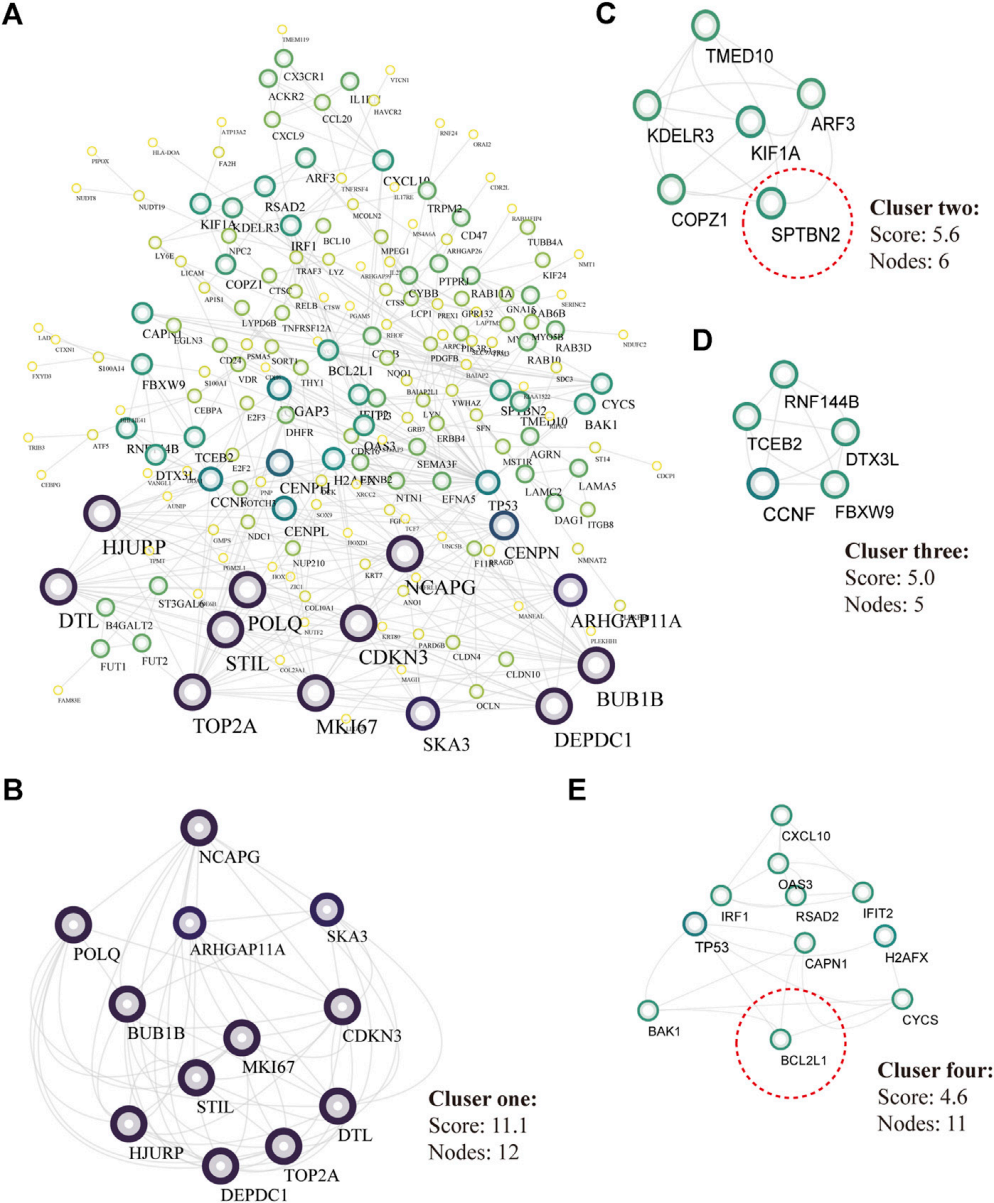
PPI analysis and core modules of the putative target genes. **(A)** PPI network analyzed by the STRING database and built by Cytoscape software (K-score >2, the circular sizes represented the degree score evaluated by MCODE plugin). **(B)** Core cluster one including 12 nodes (cutoff score = 11.1). **(C)** Core cluster two including six nodes (cutoff score = 5.6). **(D)** Core cluster one including five nodes (cutoff score = 5.0). **(E)** Core cluster one including 1,156,556 nodes (cutoff score = 4.6).

**FIGURE 7:**
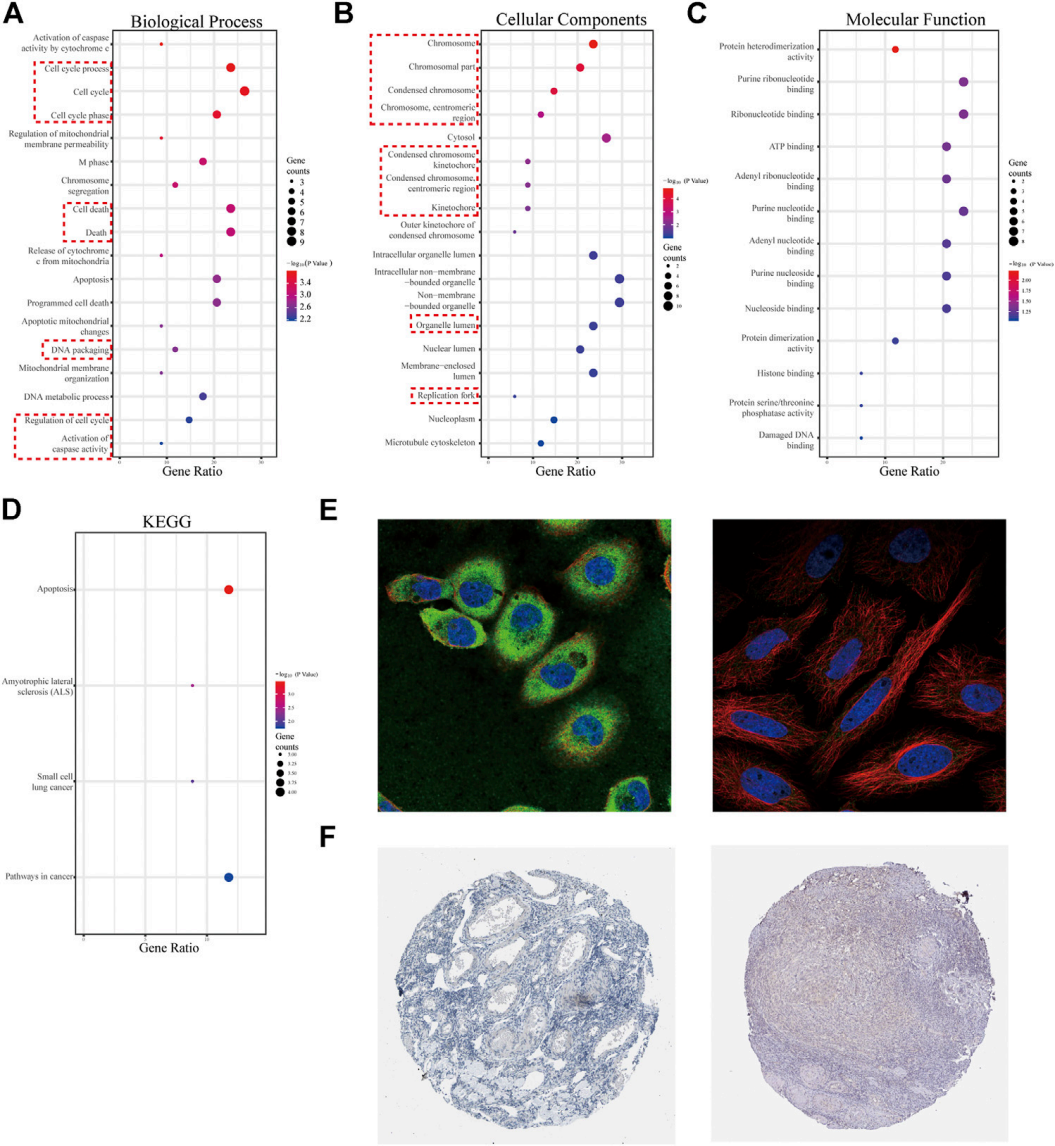
GO analysis for 34 genes from four selected modules and characteristics of the promising hub genes, *SPTBN2* and *BCL2L1*. **(A)** Biological process for the 34 genes. **(B)** Cellular components for the 34 genes. **(C)** Molecular function for the 34 genes. **(D)** KEGG pathways enriched for the 34 genes. **(E)** Cellular localization from the cell atlas of the HPA database, with SPTNB2 localizing to the cytosol and cell junctions, stained in A-431 and MCF-7 cell lines **(left)**, and BCL2L1 localizing to mitochondria, stained in MCF-7 cell line **(right)**. **(F)** Decreased expression of SPTBN2 **(left)** and BCL2L1 **(right)** in normal ovarian tissue.

There were 334 overlapped target genes selected as interactive genes with *miRNA-1827,* which were collected from 4,166 genes from the miRWALK database based on 12 servers and 2,611 overexpressed genes from the GEPIA database (Figure 5A). After the intersection, these 334 target genes were processed by bioinformatic analysis, and the results were interpreted by GO functional annotation and KEGG analysis in the DAVID database. For GO analysis, it was performed by three modules, including biological process, cellular component, and molecular function. For the biological process part, the extracellular matrix organization, cell–cell adhesion, regulation of cell proliferation and migration, actin skeleton organization, and mitochondrial membrane permeability were the main processes (Figure 5B, left panel). As for the cellular component, the cell–cell adherent junctions, extracellular exosome, transport vesicle, actin cytoskeleton, and lysosome were the primary components (Figure 5B, middle panel). Regarding the molecular function, the cadherin binding involved in cell–cell adhesion, myosin Ⅴ binding, protein kinase binding, and protein self-association were the top enriched functions (Figure 5B, right panel). Concerning KEGG pathways, cell lung cancer, hepatitis C, tight junction, and cell adhesion molecules were the major involved pathways (Figure 5C). PPI network filtered out four main modules according to their MCODE score (Figure 6). These four clusters consisted of 34 nodes or genes, as listed in Table 2. As seen, GO and KEGG enrichment of these 34 genes demonstrated that they were mainly involved in the activation of caspase activity by cytochrome c, cell cycle, and regulation of mitochondria membrane permeability for the biological process (Figure 7A). The chromosome, cytosol, and kinetochore were enriched for the cellular component (Figure 7B), and the protein heterodimerization activity, purine ribonucleotide binding, and ATP binding for molecular function were determined (Figure 7C). The pathways in cancers, tight junction, and cell adhesion molecules for KEGG were identified (Figure 7D). All the valuable information above gave us some tips and clues of the localization and molecular functions of these genes in OV, which could help us narrow the targets down to several specific hub genes.

**TABLE 2:** 34 target genes from the four clusters.

Names	Clusters	MCODE score	Names	Clusters	MCODE score	Names	Clusters	MCODE score
*NCAPG*	One	11.1	*TMED10*	Two	5.6	*CXCL10*	Four	4.6
*POLQ*	One	11.1	*KDELR3*	Two	5.6	*OAS3*	Four	4.6
*ARHGAP11A*	One	11.1	*KIF1A*	Two	5.6	*IRF1*	Four	4.6
*SKA3*	One	11.1	*ARF3*	Two	5.6	*RSAD2*	Four	4.6
*BUB1B*	One	11.1	*COPZ1*	Two	5.6	*IFIT2*	Four	4.6
*MKI67*	One	11.1	*SPTBN2*	Two	5.6	*TP5*	Four	4.6
*CDKN3*	One	11.1	*RNF144B*	Three	5.0	*CAPN1*	Four	4.6
*STIL*	One	11.1	*TCEB2*	Three	5.0	*H2AFX*	Four	4.6
*DTL*	One	11.1	*DTX3L*	Three	5.0	*BAK1*	Four	4.6
*HJURP*	One	11.1	*CCNF*	Three	5.0	*BCL2L1*	Four	4.6
*TOP2A*	One	11.1	*FBXW9*	Three	5.0	*CYCS*	Four	4.6
*DEPDC1*	One	11.1						

### Characteristics and Clinical Value of the Hub Genes

**FIGURE 8:**
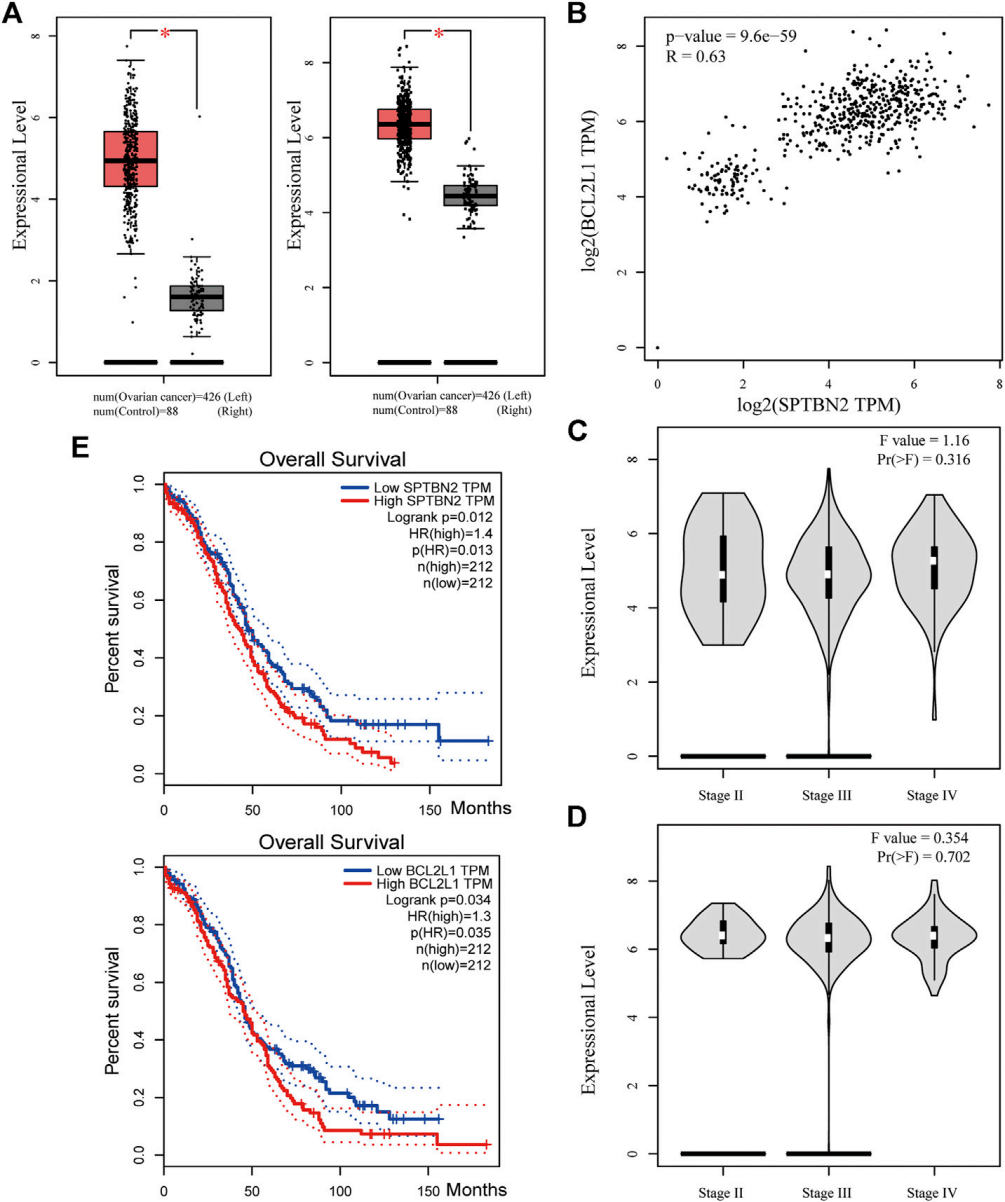
Clinical value of the two hub genes. **(A)** Expressional levels between OV and NC groups for *SPTBN2* **(left)** and *BCL2L1* **(right)** (*n* = 426 for OV or 88 for NC, *p* < 0.05). **(B)** The correlation analysis for *SPTBN2* and *BCL2L1* (*p* = 9.6E-59, *R* = 0.63). **(C)** The overall survival for patients with high or low *SPTBN2* (upper panel, log-rank *p* = 0.012, HR = 1.4) and with high or low *BCL2L1* (upper panel, log-rank *p* = 0.034, HR = 1.3). **(D)** Expression of *SPTBN2* with different stages in OV (F = 1.16, Pr(>F) = 0.316). **(E)** Expression of *SPTBN2* with different stages in OV (F = 0.354, Pr(>F) = 0.702).

**FIGURE 9:**
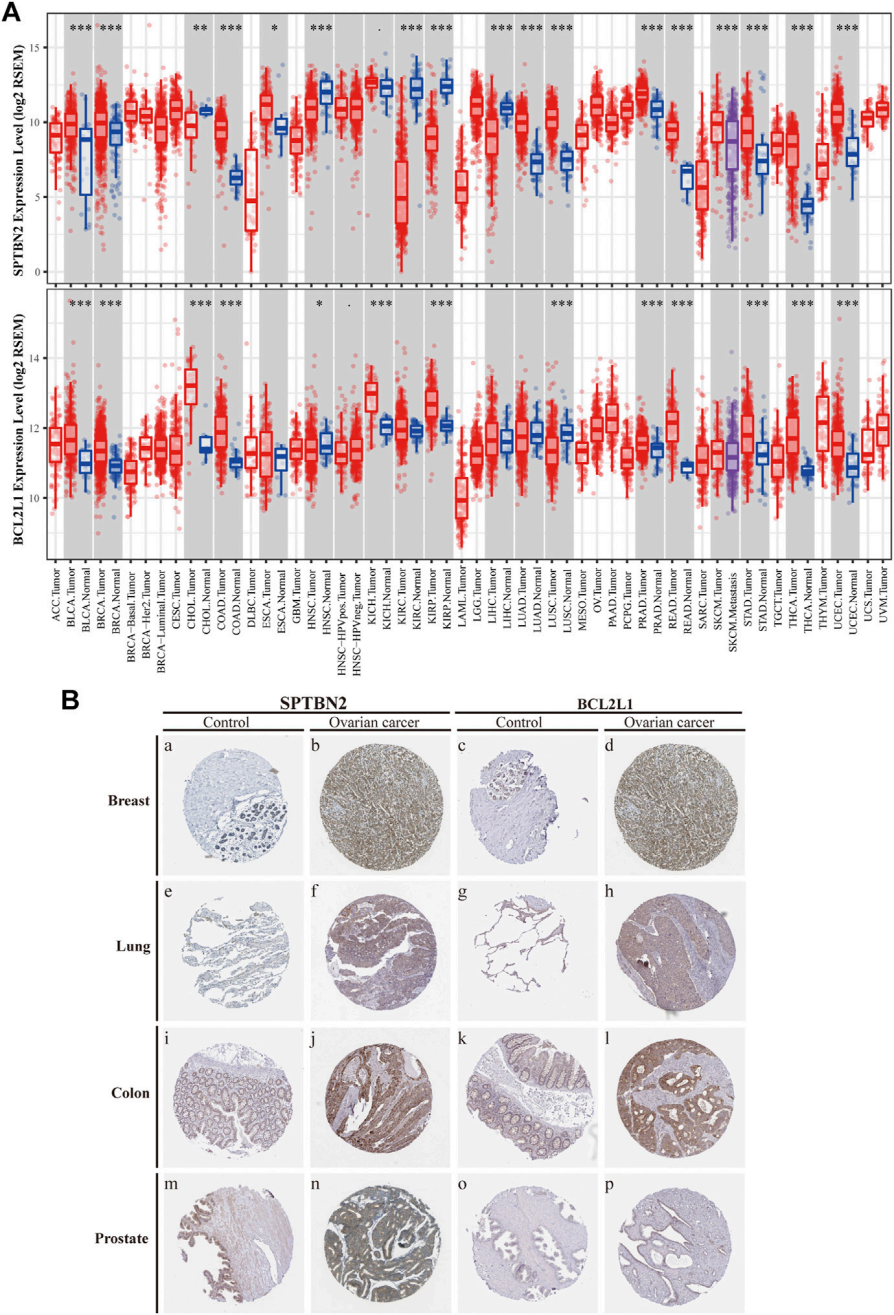
Differential expression of the two hub genes in various types of tumors compared with NC. **(A)** Distributions of gene expression levels are displayed using box plots from the TIMER database by the Wilcoxon test for *SPTBN2* **(upper panel)** and *BCL2L1* **(lower panel)**. **(B)** Differential expression at protein levels between cancers and normal tissues, verified by the protein atlas in the HPA database including the breast, lung, colon, and prostate.

According to the clinical value retrieved in the GEPIA and HPA databases in OV, two hub genes, *SPTBN2* and *BCL2L1*, caught our attention. SPTBN2 and BCL2L1 were located in the cytosol or cell junctions (SPTBN2, Figure 7E, left) and mitochondria (BCL2L1, Figure 7E, right), respectively. Also, SPTBN2 and BCL2L1 were negatively expressed in normal ovarian tissues at the protein level, as depicted by the immunohistochemical results (Figure 7F). As we could see, *SPTBN2* and *BCL2L1* were overexpressed significantly in the OV group compared with the normal controls (*p* < 0.05), as shown in Figure 8A. The elimination of the inhibitory effect of miRNAs might account for the changes with the downregulation of *miRNA-1827*. To further verify the accuracy of these results, the gene expression profiles from the ICGC database were retrieved and processed. Consistent with the above findings, the contents of *SPTBN2* and *BCL2L1* were relatively overexpressed in these 111 cases of OV patients compared with the controls (*p* < 0.01) (Supplementary Figures 2A,B). In addition, there existed a moderate correlation between the expression of *SPTBN2* and *BCL2L1* with a significant difference (*p* = 9.6E-59, Figure 8B). Furthermore, OV patients in stage Ⅱ, Ⅲ, or Ⅳ shared similar gene expression patterns for *SPTBN2* (Figure 8C) or *BCL2L1* (Figure 8D) in cancer tissues. However, the upregulation of the two hub genes would lead to an elevated risk of death, as displayed in the survival curves (Figure 8E) (*SPTBN2*, upper panel, log-rank *p* = 0.012, HR = 1.4; *BCL2L1*, lower panel, log-rank *p* = 0.034, HR = 1.3). To further investigate the hub genes and their crucial roles in various types of cancers, we detected the gene and protein expression levels among different cancers. The images for qualitative analysis in this study were obtained from the HPA database. The results demonstrated that *SPTBN2* and *BCL2L1* were relatively overexpressed in most cancers compared with normal tissues, as delineated in Figure 9A at the gene level and Figure 9B at the protein level. These results indicated that the two hub genes might be suitable biomarkers not only in OV but also in other cancers, such as breast cancer, lung cancer, colon cancer, and prostate cancer.

### The Potential Mechanisms of the Hub Genes Involved in Tumor Immunomodulatory Actions

**FIGURE 10:**
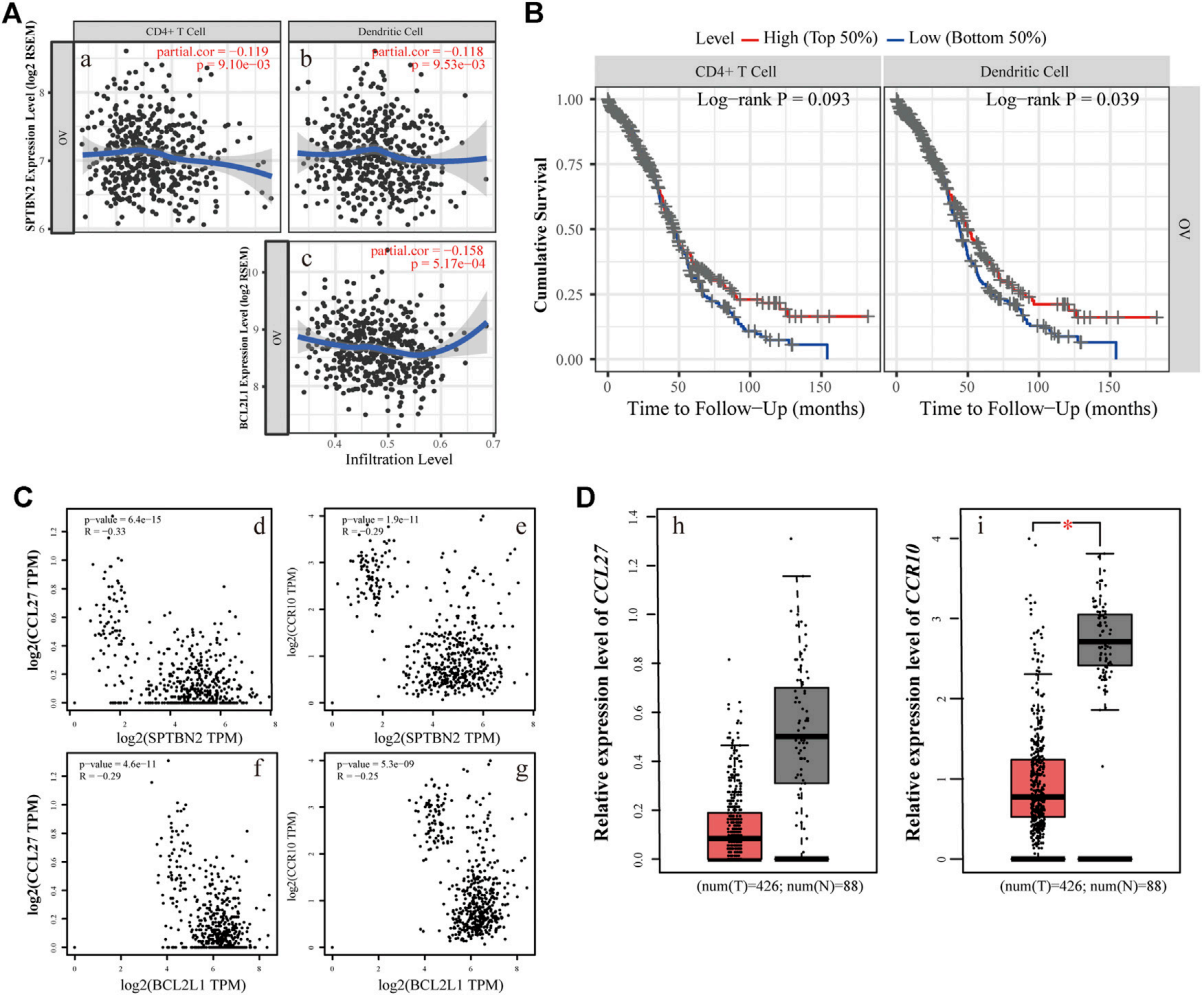
Immunoregulatory relationship of the hub genes. **(A)** The relevance of the expression level and immunocyte infiltration, where *SPTBN2* modulated CD4^+^ T cells **(i)** or dendritic cells **(ii)** and *BCL2L1* regulated dendritic cells **(iii)**. **(B)** Cumulative survival difference among OV patients with high or low CD4^+^ T cells **(left panel)** and high or low dendritic cell infiltration **(right panel)**. **(C)** The correlation between chemokine family or receptors and the hub genes. The expression levels of *CCL27* and *CCR10* negatively correlated with *SPTBN2* **(i,ii)** and *BCL2L1* **(iii,iv)**, respectively. **(D)** Differential gene expression of *CCL27* **(i)** and *CCR10* **(ii)** with 426 OV samples vs. 88 NC samples.

To further decipher the function of the hub genes in the modulation of the tumor-infiltrating immune cells, the TIMER database was used to determine the changes of immunocytes in OV tissue and their interactive relationship with the hub genes. *SPTBN2* was negatively correlated, to a certain degree, with the infiltration of CD4^+^ T cells (Figure 10Ai, partial.cor = −0.119, *p* = 9.10E-03) as well as dendritic cells (Figure 10Aii, partial.cor = −0.118, *p* = 9.53E-03). Similarly, *BCL2L1* was also negatively correlated, up to a point, with the infiltration of dendritic cells (Figure 10Aiii, partial.cor = −0.158, *p* = 5.17E-04). All those results suggested that the two hub genes might play a crucial role in adjusting CD4^+^ T cells and dendritic cells in the OV microenvironment. The higher the expression of the hub genes, the lower the infiltration of the immunocytes. Lower immunocyte infiltration of CD4^+^ T cells and dendritic cells meant poor prognosis as presented in Figure 10B. At the same time, we also explored the survival probability with the differential expression of *SPTBN2* and *BCL2L1* with the alteration of TIC abundance (CD4^+^ T cells and dendritic cells), as shown in Supplementary Figure S3. Our results demonstrated that the downregulation of *SPTBN2* in ovarian cancer cases would reduce the risk of death when they were enriched in CD4^+^ T cells (*p* < 0.05) or dendritic cells (*p* = 0.067). These results were in line with the above findings. However, no significant difference was observed in patients with the differential expression of *BCL2L1* with the altered infiltration of dendritic cells. For further research on the chemokines related to the immunocytes and the two hub genes, the expression and correlation of *CCL27* and *CCR10* were explored. The results demonstrated that the overexpression of *SPTBN2* had a negative correlation with the expression of *CCL27* (Figure 10Ci, *p* = 6.4E-15, *R* = −0.33) and *CCR10* (Figure 10Cii, *p* = 1.9E-11, *R* = −0.29). Likewise, *BCL2L1* was negatively correlated with the expression of *CCL27* (Figure 10Ciii, *p* = 4.6E-11, *R* = −0.29) and *CCR10* (Figure 10Civ, *p* = 5.3E-09, *R* = −0.25). The expression profile of these molecules, *CCL27* and *CCR10*, indicated downregulated trends in OV tissue, as shown in Figures 10Di,ii, for each.

## Discussion

With the advent of high-throughput sequencing and microarray technologies, a striking upsurge in identifying novel biomarkers and the construction of molecular networks in various diseases is in the making based on the advances of bioinformatic analyses (Udhaya Kumar et al., 2020b; Mishra et al., 2021). At the same time, a growing body of evidence from gene expression profiles and microarrays has confirmed the significance of miRNAs in the tumorigenesis and progression of various types of cancers (Rupaimoole and Slack, 2017). The comparatively deep explorations on their roles have promoted the identification of novel biomarkers and application of these potential intervention targets toward the era of miRNA therapeutics (Hayes et al., 2014).

Previous studies have revealed that *miRNA-1827* functions as a tumor suppressor in various types of cancers since it is involved in tumor proliferation, invasion, migration, and angiogenesis (Ho et al., 2018). However, to date, no tangible proof has ever uncovered the relationship between *miRNA-1827* and its clinical significance in OV. Here, we performed systemic bioinformatic analyses to determine its pivotal role in OV and its intrinsic targets. The highlights of this study are the inclusion of a larger sample size by pooling similar studies and the application of bioinformatics and computational biology for information mining to determine the expression level of *miRNA-1827*. As reported, *miRNA-1827* is downregulated in most cancer samples, such as human lung adenocarcinoma cells and lymphoblastic leukemia clinical cases or cell lines, and the decreased level is correlated with tumor grade and lymph node metastasis (Guo et al., 2020; Naderi et al., 2020). In line with these findings, our research has also observed the reduced expression of *miRNA-1827* in all OV cases. The significantly differential expression was further confirmed by the comprehensive meta-analysis and qRT-PCR detection of seven types of OV cell lines or normal ovarian epithelial cells. Furthermore, a significant difference was noticed in the proliferation of OV cells after transfection of the *miRNA-1827* mimic. As previously described, *miRNA-1827* has been proved to suppress the development of lung adenocarcinoma by targeting oncogenic genes *MYC* and *FAM83F* (Fan et al., 2020). Another study also confirmed that a single-nucleotide polymorphism within the binding site of *miRNA-1827* on the 3’ UTR of *MYCL1* could repress the expression level of the latter, triggering the progression of small-cell lung cancer (Xiong et al., 2011). These results signified that the overexpression of *miRNA-1827* could also act as a potential intervention pattern of OV. Subsequently, 334 candidate interactive genes targeted by *miRNA-1827* were screened out based on the miRWALK and GEPIA databases; GO and KEGG enrichment analyses were carried out to determine the characteristics and functions of these genes. According to the results, the most meaningful finding that emerged from the analyses was that these targets of miRNAs were involved in the regulation of cell proliferation and migration, extracellular matrix organization, cell–cell adhesion, actin skeleton organization, and actin filament bundle assembly. Intriguingly, *miRNA-1827* has been known to engage in biological activities chiefly, including the modulation of cellular proliferation, invasion, and metastasis in reproductive or nonproductive tumors (Wang et al., 2020; Shen et al., 2021). It is worth noting that altered cytoskeleton-associated proteins have been proven to produce an effect on the clinical outcomes of OV patients during malignant progression (Schiewek et al., 2018). In addition to this, cytoskeletal dynamics, such as those of the actin filament, play a pivotal role in enhanced cell motility to promote OV metastasis through epithelial–mesenchymal transition (Lee et al., 2013). To elucidate the hub interactions among PPI networks, the MCODE plugin in Cytoscape software was applied, and finally, 34 node genes were identified. These genes were the main components of the chromosome (centromeric region), kinetochore, organelle lumen, and replication fork. As expected, these genes exerted considerable influence on the cell cycle process, chromosome segregation, DNA packaging, activation of caspase activity, and cell death. Based on this, two hub genes, *SPTBN2* and *BCL2L1*, were determined as target genes of *miRNA-1827* for further research according to the clinical value retrieved in the GEPIA and HPA databases in OV. *SPTBN2* is a protein-encoding gene, and its protein product belongs to the spectrin family. *SPTBN2* was previously reported in the development of cognition and motion or neurologic disorders (Forman et al., 2012; Lise et al., 2012). Additionally, it was recently identified as a signature gene, emerging in seven common cancers in the pathogenesis of cancers and pattern recognition (Wen et al., 2018). In the present study, *SPTBN2* was confirmed for the first time to be upregulated in OV at gene and protein levels. Most studies mainly focused on the role of *SPTBN2* in neurogenesis and degenerative diseases as a structural carrier for the stabilization and activation of membrane channels, receptors, and transporters (Yildiz Bolukbasi et al., 2017; Louis and Faust, 2020). In terms of cancer research, it was identified to be associated with the tumor progression and survival of colorectal cancer patients with m6A modifications (Zhang et al., 2021). Besides, it has also been reported to be a member of a novel, competing, endogenous RNA network for the prognosis of bladder cancer (Zhang et al., 2021). As for *BCL2L1*, it is an anti-apoptotic regulator of the *BCL-2* family and causes momentous effects on the integrity of the mitochondria membrane, autophagy, and cell survival (Duan et al., 2019). *BCL2L1* has made significant differences in tumor targeting therapy, as a novel tumor promoter, for hepatocellular carcinoma, lung cancer, gastric cancer, and cervical cancer (Park et al., 2015; Cai et al., 2017). Here, we found that *BCL2L1* was also overexpressed in OV specimens. As a pro-oncogene, the aberrant expression of *BCL2L1* is related to a shortened disease-free survival, which accrues in patients with recurrence following chemotherapeutic interventions in OV (Williams et al., 2005). Preclinical research has also demonstrated that the ectopic expression of *BCL2L1* confers resistance to various pharmaceutical products, such as cisplatin, vincristine, and gemcitabine (Schniewind et al., 2004). Posttranslational modification by deamidation of *BCL2L1* has also been proven to be implicated in drug resistance to DNA-damaging medicaments (Deverman et al., 2002), and the pro-survival action of the oncogenic tyrosine kinase LCK following DNA damage may be mediated partly by restraining deamidation of *BCL2L1* as well (Zhao et al., 2004). All these findings have therefore specified the promising therapy of targeting *BCL2L1* in OV management. Besides, our investigation showed that these two hub genes were co-expressed and correlated with each other. The overexpression of these molecules would increase the risk of death or give rise to a poorer prognosis. During the progression of OV, they were persistently highly expressed across stages Ⅱ, Ⅲ, and Ⅳ. It should be mentioned that *SPTBN2* and *BCL2L1* were upregulated together not only in OV but also in various types of cancers, which were validated by various types of tumors and normal samples at both the gene and protein levels. Overall, these discoveries suggested the prognostic significance of *SPTBN2* and *BCL2L1* as therapeutic biomarkers targeted by *miRNA-1827* in OV.

In the following exploration, we have focused on the roles of *SPTBN2* and *BCL2L1* in the tumor microenvironment from the perspective of immune modulation by retrieving data from the TIMER database. Existing studies demonstrate that the interaction between tumor cells and host cells is indispensable for the oncogenesis and progression of tumors (Grivennikov et al., 2010). Within this environment and upon tumor-driven stimuli, tumors can form a tumor-permissive soil with reprogrammed host cells that exhibit tumor-supporting phenotypes (Binnewies et al., 2018). Intriguingly, this study revealed that *SPTBN2* and *BCL2L1* could generate adverse effects on the decreased infiltration of CD4^+^ T cells and dendritic cells in OV, which seemed likely to bring about a more unsatisfactory prognostic outcome. The reduced abundance of CD4^+^ T cells and dendritic cells in this study was confirmed to be associated with declined cumulative survival rate. In order to seek the reasons for the reduction of CD4^+^ T cells and dendritic cells mediated by *SPTBN2* or *BCL2L1*, the related chemokine *CCL27* and the receptor *CCR10* were explored. In terms of the traits of the two molecules, *CCL27*, as a member of the chemokine ligand family, would participate in the homing of immunocytes by specifically binding to *CCR10* (Fujita et al., 2006). *CCL27* was once regarded as a sensitive serum biomarker to distinguish nasopharyngeal carcinoma patients from healthy donors (highly expressed in abnormal populations due to the activated immune system) (Mao et al., 2018). As reported previously, *CCL27* could inhibit tumor growth to a certain extent through recruiting natural killer cells or T cells locally (Gao et al., 2003) and could intensify the resistance to tumor formation as well (Okada et al., 2004). In addition, *CCL27* could enter into a chemotaxis role for the recruitment and activation of CD4^+^ T cells. Our study found that these molecules were also negatively influenced by *SPTBN2* or *BCL2L1*. In accordance with our studies, people once reported (Khaiboullina et al., 2015) that the reduced chemokine of *CCL27* could help cancers to evade immunological surveillance, thereby resulting in immune escape (Pivarcsi et al., 2007). The results above delineate the alteration of *SPTBN2* and *BCL2L1* in view of tumor immune modulation mediated by *CCL27* and *CCR10*, resulting in a reduced abundance of CD4^+^ T cells and dendritic cells.

The limitations of the current study need to be pointed out: the mutual interactions between *miRNA-1827* and its two hub genes should be further validated to ensure the target-binding action. Besides, the effects of *miRNA-1827* in OV should be verified in animal models to promote its clinical application.

## Conclusion

In conclusion, the present study takes advantage of retrieved data from microarrays or gene expression profiles and integrative bioinformatic analyses. Therefore, we have identified that *miRNA-1827* as a novel biomarker is associated with the proliferation and prognosis of OV by targeting *SPTBN2* and *BCL2L1*. These hub genes are determined to be involved in the tumor immunomodulatory process mediated by *CCL27* and *CCR10*. On the whole, *miRNA-1827* from computational biology analysis exerts promising effects as an intervention target in OV management in future applications.

## Data Availability

The datasets presented in this study can be found in online repositories. The names of the repository/repositories and accession number(s) can be found below: GSE119056, GSE83693, GSE53829, GSE47841, GSE48485, and GSE58517.
