# Supplementary figures and images for: A Comprehensive Analysis of the Downregulation of miRNA-1827 and Its Prognostic Significance by Targeting SPTBN2 and BCL2L1 in Ovarian Cancer
Source: Front Mol Biosci. 2021 Jun 11;8:687576. doi: 10.3389/fmolb.2021.687576 (PMC8226272; doi:10.3389/fmolb.2021.687576)

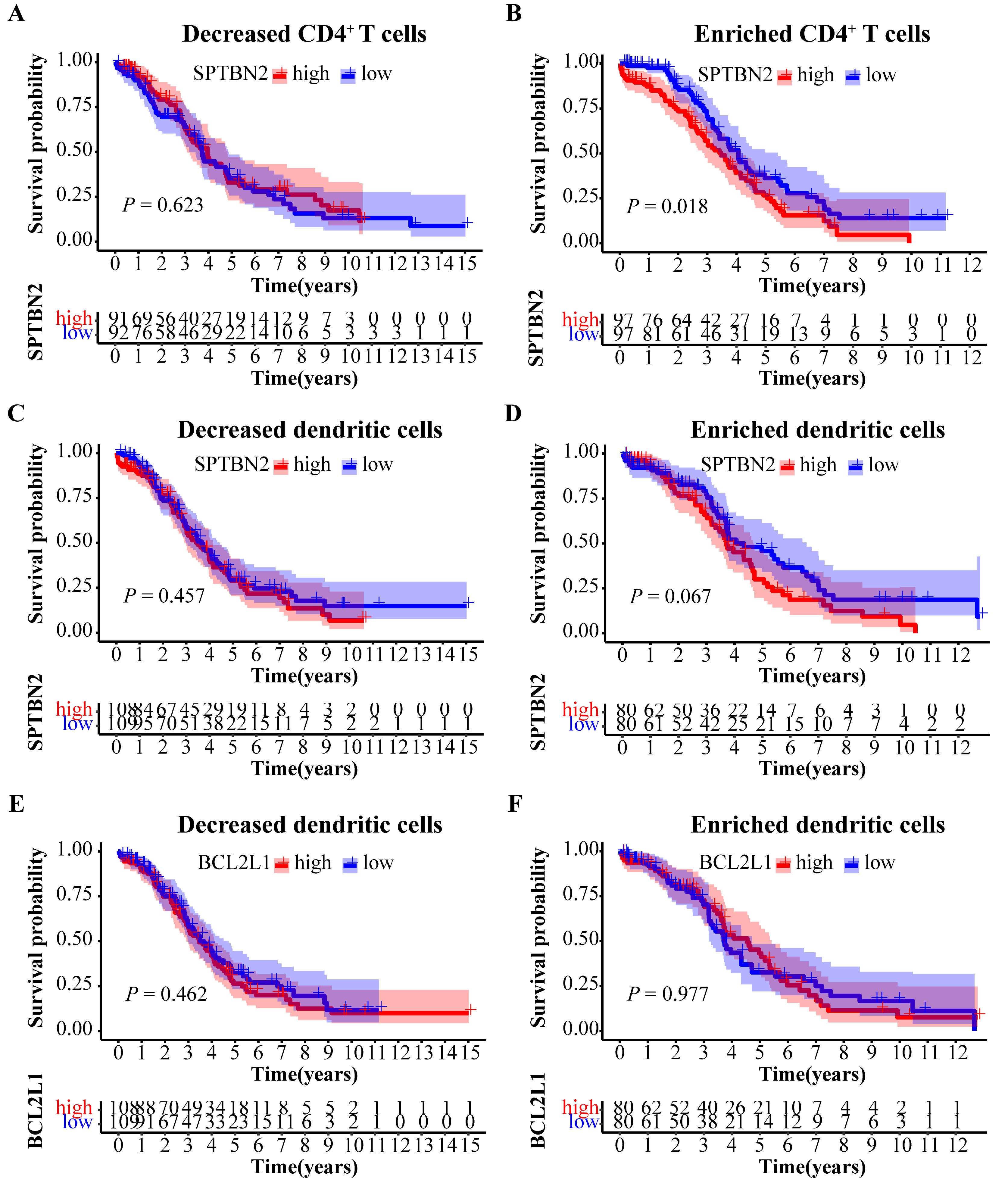

Supplement: Supplementary file 1 [file Image3.TIF]

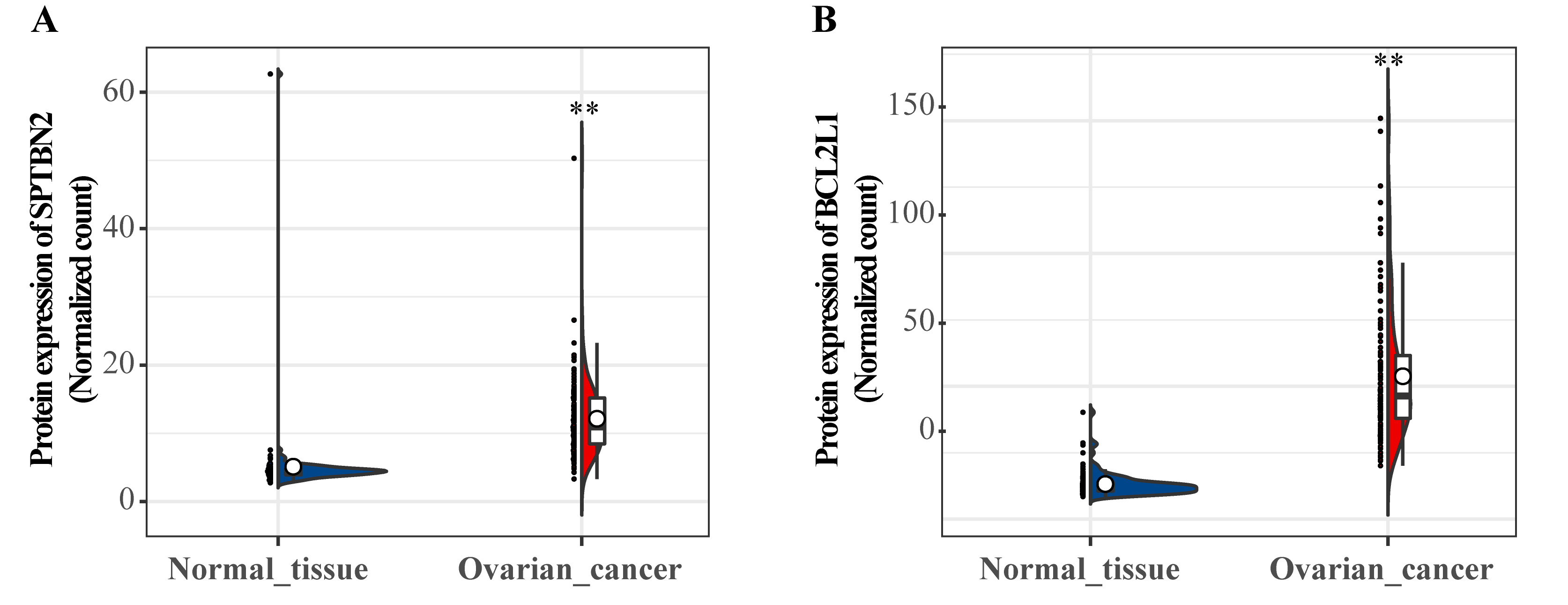

Supplement: Supplementary file 2 [file Image2.TIF]

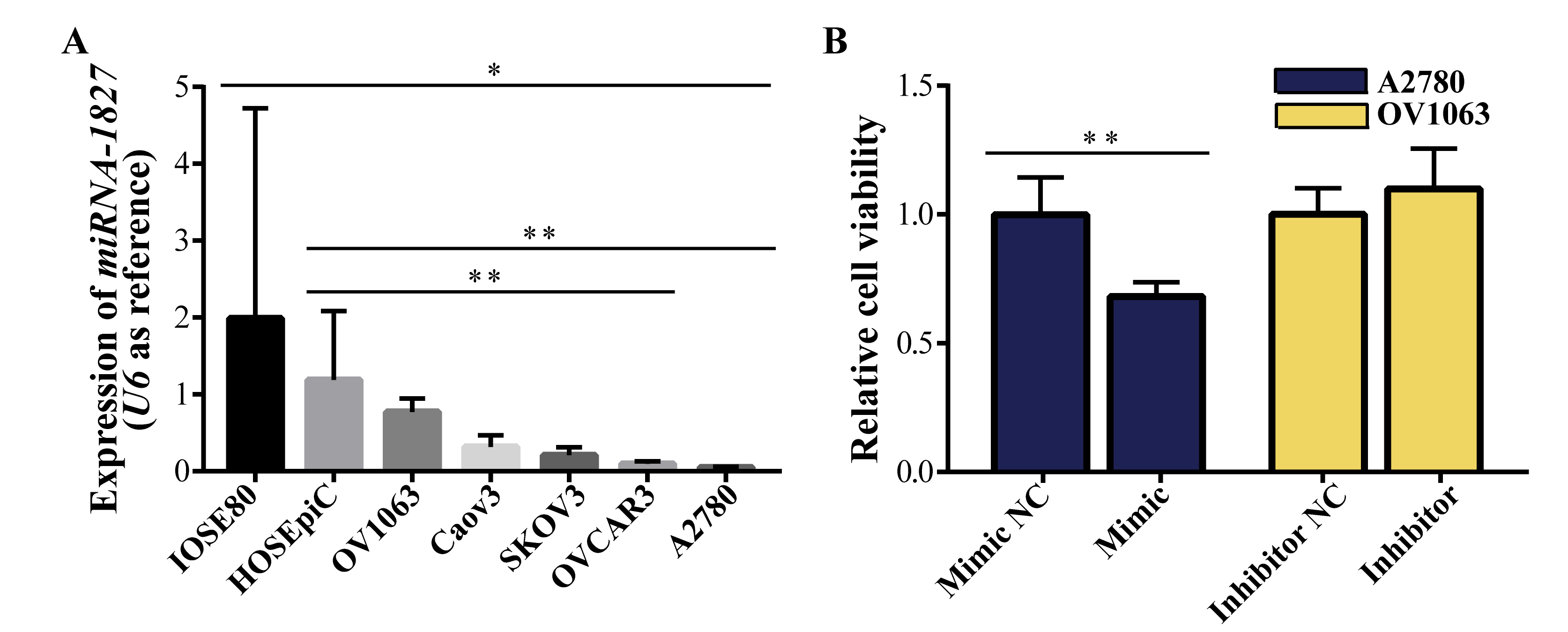

Supplement: Supplementary file 3 [file Image1.TIF]
