# Supplementary material for: A Comprehensive Analysis of the Downregulation of miRNA-1827 and Its Prognostic Significance by Targeting SPTBN2 and BCL2L1 in Ovarian Cancer
Source: Front Mol Biosci. 2021 Jun 11;8:687576. doi: 10.3389/fmolb.2021.687576 (PMC8226272; doi:10.3389/fmolb.2021.687576)
Supplement: Supplementary file 4 [file Table1.DOC]

**Supplementary Table 1.** 334 differentially expressed genes shared by the miRWALK and GEPIA databases.

| **Gene names** | **Log fold change** | **Adjusted *P*-value** |
| --- | --- | --- |
| *TRIB3* | 1.463 | 1.59E-22 |
| *IPO9* | 1.05 | 2.54E-28 |
| *TMEM19* | 1.615 | 2.86E-40 |
| *CHRAC1* | 1.674 | 9.67E-48 |
| *CORO2A* | 2.532 | 7.71E-81 |
| *ST8SIA4* | 1.034 | 5.96E-25 |
| *TPM3* | 2.095 | 5.04E-75 |
| *MAP3K13* | 1.401 | 8.30E-35 |
| *KIAA1324* | 1.3 | 1.09E-14 |
| *MMP15* | 3.162 | 5.95E-83 |
| *CAPN1* | 1.087 | 4.02E-29 |
| *MYO5C* | 1.28 | 2.09E-29 |
| *MCOLN2* | 1.031 | 2.91E-19 |
| *PCCB* | 1.679 | 4.15E-51 |
| *TDRKH* | 1.844 | 2.31E-55 |
| *SLC9A3R1* | 2.527 | 9.97E-66 |
| *NMNAT2* | 1.08 | 2.20E-18 |
| *IFITM10* | 1.194 | 1.58E-29 |
| *HOXB5* | 1.616 | 1.71E-17 |
| *SOX9* | 4.174 | 6.07E-89 |
| *GRHL1* | 1.505 | 1.93E-31 |
| *CENPL* | 1.493 | 1.68E-47 |
| *HLA-DOA* | 2.145 | 3.32E-20 |
| *LIMD2* | 1.24 | 3.78E-19 |
| *TMEM189* | 1.212 | 1.25E-33 |
| *MYCL* | 3.675 | 5.40E-68 |
| *RAB11FIP4* | 2.107 | 4.15E-39 |
| *CLDN10* | 4.208 | 8.96E-42 |
| *NOL4L* | 2.713 | 5.17E-95 |
| *ARHGAP26* | 1.11 | 6.44E-19 |
| *CDK16* | 1.06 | 2.95E-17 |
| *AIF1L* | 2.81 | 1.62E-57 |
| *GGT6* | 3.04 | 4.83E-63 |
| *MALL* | 1.307 | 1.19E-12 |
| *MAGI1* | 1.009 | 3.50E-17 |
| *PSMA5* | 1.067 | 1.01E-32 |
| *DEPDC1* | 2.235 | 2.98E-70 |
| *NUTF2* | 1.314 | 3.42E-47 |
| *KRT80* | 2.851 | 7.65E-47 |
| *AGRN* | 3.548 | 3.27E-90 |
| *OCLN* | 1.986 | 2.97E-58 |
| *ATP13A2* | 1.224 | 4.61E-31 |
| *NUDT8* | 1.189 | 3.68E-26 |
| *PSD4* | 2.93 | 3.24E-106 |
| *TMC5* | 1.38 | 5.14E-26 |
| *NUDT19* | 1.088 | 6.35E-32 |
| *DBNDD1* | 2.315 | 1.17E-55 |
| *SCARA3* | 2.559 | 7.00E-31 |
| *PLEKHH1* | 1.199 | 1.37E-23 |
| *GDF11* | 1.082 | 4.57E-08 |
| *SPTBN2* | 3.338 | 4.95E-86 |
| *SDC3* | 2.148 | 1.16E-33 |
| *E2F2* | 1.748 | 7.35E-60 |
| *TMUB1* | 1.148 | 3.95E-40 |
| *ESRP1* | 5.719 | 1.06E-179 |
| *CXCL10* | 5 | 1.17E-55 |
| *KIF1A* | 2.574 | 2.32E-16 |
| *FAM124A* | 1.568 | 4.87E-31 |
| *SLC6A12* | 1.663 | 3.29E-23 |
| *MGAT3* | 1.67 | 1.48E-27 |
| *PTPRJ* | 1.399 | 2.68E-34 |
| *MPEG1* | 1.364 | 2.69E-14 |
| *CCNF* | 1.6 | 1.99E-43 |
| *NMT1* | 1.354 | 2.32E-22 |
| *SEMA3F* | 3.497 | 1.53E-68 |
| *PREX1* | 1.05 | 3.77E-16 |
| *RAB10* | 1.08 | 8.39E-25 |
| *CX3CR1* | 1.367 | 1.08E-23 |
| *FUT2* | 1.224 | 1.40E-19 |
| *TSFM* | 1.054 | 2.10E-28 |
| *BAIAP2* | 1.569 | 1.28E-40 |
| *PARD6B* | 2.024 | 3.92E-83 |
| *AP1S1* | 1.056 | 3.51E-42 |
| *PNP* | 2.375 | 7.94E-47 |
| *DUSP8* | 1.421 | 6.76E-14 |
| *BAIAP2L1* | 3.228 | 2.54E-111 |
| *XRCC2* | 1.29 | 8.13E-50 |
| *ERBB4* | 1.578 | 3.27E-30 |
| *THEM6* | 2.835 | 1.94E-88 |
| *MPPED2* | 2.439 | 1.16E-36 |
| *IRF1* | 1.433 | 1.21E-16 |
| *UNC5B* | 1.506 | 2.16E-20 |
| *RHOF* | 2.464 | 3.10E-45 |
| *KIAA1522* | 1.539 | 3.49E-28 |
| *SYNGR3* | 1.223 | 1.15E-19 |
| *MYO6* | 1.049 | 1.18E-15 |
| *PLEKHB1* | 3.235 | 4.20E-61 |
| *GRB7* | 4.794 | 8.48E-128 |
| *DDAH1* | 1.819 | 2.81E-51 |
| *CTSW* | 1.123 | 6.96E-13 |
| *TRAF3* | 1.079 | 9.48E-20 |
| *FAT2* | 1.457 | 1.44E-26 |
| *PRRG4* | 2.208 | 2.67E-67 |
| *LRRC25* | 1.09 | 8.62E-17 |
| *MS4A6A* | 1.298 | 0.000000582 |
| *ARPC5* | 1.731 | 7.27E-47 |
| *SYNGR2* | 2.198 | 5.48E-60 |
| *EMID1* | 1.32 | 5.92E-12 |
| *MOCS3* | 1.12 | 1.98E-42 |
| *FA2H* | 1.336 | 2.98E-35 |
| *STC1* | 1.35 | 2.74E-18 |
| *PDGFB* | 1.024 | 8.89E-16 |
| *TMEM209* | 1.041 | 7.37E-30 |
| *FAM83E* | 1.771 | 3.22E-27 |
| *ST3GAL6* | 1.345 | 3.12E-22 |
| *FBXW9* | 1.325 | 2.40E-30 |
| *NINJ2* | 2.518 | 1.52E-40 |
| *MRPL35* | 1.19 | 1.99E-50 |
| *CDH6* | 3.847 | 5.09E-45 |
| *SFN* | 3.792 | 4.11E-62 |
| *CENPN* | 2.256 | 9.01E-65 |
| *LYPLA2* | 1.077 | 1.03E-24 |
| *S100A1* | 7.86 | 4.29E-105 |
| *H2AFX* | 1.961 | 3.14E-60 |
| *KDELR3* | 2.558 | 1.74E-57 |
| *PLEKHF2* | 1.216 | 2.49E-28 |
| *HOXD1* | 2.782 | 4.23E-28 |
| *POLQ* | 1.177 | 1.35E-42 |
| *TNFRSF12A* | 4.099 | 3.01E-98 |
| *C1QL4* | 1.077 | 2.33E-15 |
| *NUP210* | 2.853 | 1.52E-70 |
| *BCAS4* | 1.569 | 1.04E-32 |
| *HSD3B7* | 1.206 | 1.41E-19 |
| *VDR* | 2.067 | 4.75E-43 |
| *PODNL1* | 1.5 | 2.62E-16 |
| *L1CAM* | 2.71 | 1.49E-26 |
| *SKA3* | 2.877 | 2.61E-106 |
| *SH3RF2* | 1.313 | 4.96E-25 |
| *MST1R* | 1.21 | 2.34E-13 |
| *PGAM5* | 1.698 | 2.56E-49 |
| *FUT1* | 1.075 | 5.54E-44 |
| *NCS1* | 1.007 | 7.50E-17 |
| *LYN* | 1.995 | 1.40E-41 |
| *NLN* | 1.174 | 8.87E-31 |
| *LY75* | 2.115 | 1.36E-53 |
| *STIL* | 1.795 | 3.75E-59 |
| *NQO1* | 2.721 | 2.01E-41 |
| *RAB3D* | 2.131 | 5.45E-53 |
| *RNF144B* | 2.138 | 4.76E-44 |
| *NTN1* | 2.001 | 3.25E-32 |
| *TOP2A* | 4.32 | 6.03E-116 |
| *EFNB2* | 1.41 | 1.29E-17 |
| *CEBPG* | 1.142 | 3.12E-26 |
| *SHROOM3* | 2.738 | 4.73E-67 |
| *PLAGL2* | 1.209 | 8.42E-19 |
| *LRRC59* | 1.16 | 5.41E-26 |
| *ST14* | 5.928 | 1.54E-156 |
| *CEBPA* | 2.139 | 9.60E-40 |
| *SLC12A5* | 3.041 | 2.22E-44 |
| *KIF24* | 1.142 | 4.80E-35 |
| *C1ORF226* | 1.416 | 1.76E-34 |
| *ORAI2* | 1.191 | 8.98E-18 |
| *PPP1R16B* | 1.133 | 4.88E-15 |
| *TNFAIP2* | 2.681 | 2.98E-44 |
| *MMD* | 1.082 | 4.26E-14 |
| *TCEB2* | 1.239 | 5.29E-33 |
| *RAB11A* | 1.034 | 3.87E-28 |
| *VANGL1* | 2.019 | 1.95E-52 |
| *EGLN3* | 2.39 | 5.21E-30 |
| *RRAGD* | 1.432 | 1.99E-22 |
| *ZNF28* | 1.062 | 8.96E-30 |
| *TCF7* | 1.598 | 1.95E-31 |
| *THSD4* | 2.995 | 4.63E-31 |
| *TMEM119* | 1.583 | 8.63E-17 |
| *NOTCH3* | 1.407 | 1.63E-11 |
| *CDKN3* | 3.296 | 3.16E-98 |
| *E2F3* | 1.916 | 8.85E-57 |
| *NDC1* | 1.459 | 1.88E-44 |
| *PYCRL* | 1.365 | 5.29E-39 |
| *STK17B* | 1.807 | 3.19E-44 |
| *ADD2* | 1.826 | 6.18E-29 |
| *TMEM139* | 4.789 | 4.37E-129 |
| *CYCS* | 1.405 | 2.33E-30 |
| *IGF2BP3* | 1.38 | 1.79E-17 |
| *BAK1* | 1.889 | 1.70E-59 |
| *LRRN2* | 3.195 | 1.15E-52 |
| *CXADR* | 3.495 | 4.19E-107 |
| *ZNF48* | 1.31 | 6.63E-39 |
| *ARHGAP39* | 1.846 | 3.28E-50 |
| *RIPK4* | 3.017 | 2.39E-82 |
| *NPC2* | 1.079 | 4.84E-17 |
| *ADGRF1* | 1.389 | 2.61E-27 |
| *SORT1* | 2.984 | 8.26E-75 |
| *LLGL2* | 1.671 | 1.90E-41 |
| *PCDH7* | 1.937 | 1.03E-30 |
| *TMEM92* | 1.885 | 4.41E-26 |
| *TMEM33* | 1.521 | 3.40E-38 |
| *VTCN1* | 5.003 | 2.00E-60 |
| *PAEP* | 2.129 | 2.64E-17 |
| *DHFR* | 1.502 | 2.32E-41 |
| *GPR132* | 1.027 | 3.21E-21 |
| *TPMT* | 1.519 | 9.14E-51 |
| *YWHAZ* | 1.26 | 1.25E-23 |
| *PGM2L1* | 1.026 | 5.07E-21 |
| *CTSB* | 1.535 | 1.75E-27 |
| *KIAA1161* | 2.116 | 2.23E-40 |
| *COL23A1* | 2.161 | 3.10E-32 |
| *CYB561* | 1.5 | 3.46E-34 |
| *THY1* | 3.448 | 1.41E-39 |
| *TMPRSS13* | 1.581 | 1.39E-24 |
| *TRPM2* | 2.111 | 2.12E-54 |
| *CNFN* | 3.528 | 9.93E-59 |
| *ITGB8* | 1.944 | 1.52E-30 |
| *CDR2L* | 2.858 | 1.01E-56 |
| *ACKR2* | 1.075 | 7.84E-17 |
| *MPDU1* | 1.294 | 7.63E-35 |
| *DAG1* | 1.38 | 1.74E-29 |
| *PI3* | 4.624 | 2.60E-42 |
| *BNIPL* | 1.582 | 6.89E-15 |
| *LYPD6B* | 5.139 | 4.11E-102 |
| *IQGAP3* | 2.578 | 3.33E-74 |
| *EFNA5* | 1.212 | 3.71E-11 |
| *NXN* | 1.388 | 1.34E-22 |
| *HN1L* | 1.6 | 2.01E-37 |
| *ICMT* | 1.325 | 4.66E-28 |
| *UQCC3* | 1.051 | 2.95E-40 |
| *SLC35E1* | 1.105 | 1.08E-21 |
| *LAD1* | 4.654 | 4.15E-128 |
| *SRD5A3* | 3.275 | 2.35E-82 |
| *CYBB* | 1.644 | 1.85E-17 |
| *C11ORF45* | 1.123 | 8.72E-36 |
| *C2* | 3.097 | 4.53E-43 |
| *SLC43A2* | 1.454 | 9.07E-26 |
| *PDE6B* | 1.499 | 3.96E-26 |
| *MRPS16* | 1.184 | 3.95E-36 |
| *KCMF1* | 1.136 | 3.70E-22 |
| *RNF24* | 1.354 | 1.80E-26 |
| *SBK1* | 2.609 | 2.46E-50 |
| *TMED10* | 1.204 | 2.24E-21 |
| *IL1RN* | 2.934 | 9.98E-58 |
| *KRT7* | 7.425 | 3.60E-159 |
| *NACC1* | 2.695 | 3.65E-68 |
| *SEC62* | 1.1 | 8.59E-22 |
| *MKI67* | 3.282 | 2.41E-80 |
| *TP53* | 1.228 | 0.0000596 |
| *RELB* | 1.479 | 2.54E-30 |
| *CHURC1-FNTB* | 2.232 | 1.62E-23 |
| *SLC7A1* | 2.011 | 3.76E-43 |
| *LAMC2* | 3.724 | 4.23E-83 |
| *LYZ* | 2.735 | 4.78E-25 |
| *LCP1* | 2.188 | 7.68E-31 |
| *CD47* | 2.62 | 2.21E-68 |
| *NUAK2* | 3.754 | 2.10E-109 |
| *UBALD2* | 2.964 | 2.27E-96 |
| *BCL10* | 1.22 | 6.68E-44 |
| *AMOT* | 1.369 | 5.29E-15 |
| *HAVCR2* | 1.598 | 1.89E-25 |
| *BHLHE41* | 3.613 | 3.83E-51 |
| *PIPOX* | 1.102 | 8.14E-17 |
| *ARHGAP11A* | 1.809 | 1.94E-53 |
| *DCK* | 1.142 | 1.16E-31 |
| *NETO2* | 1.126 | 1.17E-15 |
| *SSH2* | 1.22 | 2.18E-34 |
| *PROM2* | 3.334 | 7.74E-47 |
| *C6ORF223* | 1.606 | 6.18E-31 |
| *CLDN4* | 7.512 | 6.91E-204 |
| *PIGZ* | 1.131 | 2.34E-20 |
| *SAMD12* | 1.632 | 1.01E-56 |
| *MEOX1* | 1.87 | 7.65E-19 |
| *SGPL1* | 1.443 | 1.08E-37 |
| *STEAP3* | 1.047 | 3.03E-11 |
| *PAK6* | 1.747 | 2.63E-46 |
| *DTX3L* | 1.399 | 4.13E-29 |
| *MYO5B* | 3.554 | 5.31E-108 |
| *CENPH* | 1.148 | 6.81E-30 |
| *RAP1GAP* | 2.601 | 4.95E-52 |
| *MYEF2* | 1.983 | 9.11E-50 |
| *COPZ1* | 1.218 | 4.01E-38 |
| *CACNG4* | 3.4 | 2.88E-46 |
| *CTXN1* | 4.304 | 4.09E-94 |
| *IL17RE* | 1.444 | 9.60E-26 |
| *IFIT2* | 1.158 | 2.18E-14 |
| *PIK3R3* | 1.274 | 8.91E-22 |
| *SERINC2* | 5.068 | 2.36E-112 |
| *NDUFA4L2* | 1.2 | 3.76E-08 |
| *TNFRSF4* | 1.043 | 9.74E-25 |
| *RPUSD1* | 1.375 | 1.77E-46 |
| *OAS3* | 2.389 | 5.49E-35 |
| *CTSS* | 2.249 | 1.53E-24 |
| *ANO1* | 1.32 | 4.27E-13 |
| *ZIC1* | 1.423 | 3.02E-10 |
| *CRB2* | 2.262 | 1.38E-33 |
| *SOX12* | 1.555 | 4.65E-34 |
| *S100A14* | 4.836 | 1.33E-91 |
| *MMACHC* | 1.397 | 6.82E-51 |
| *B4GALT2* | 1.185 | 3.19E-27 |
| *RAB6B* | 2.163 | 2.98E-38 |
| *CCL20* | 2.375 | 3.10E-29 |
| *GNA15* | 1.139 | 1.14E-16 |
| *ATF5* | 1.825 | 1.75E-36 |
| *DDA1* | 1.333 | 1.62E-39 |
| *MANEAL* | 1.36 | 3.61E-30 |
| *TES* | 2.679 | 8.31E-63 |
| *LAMA5* | 1.934 | 7.78E-33 |
| *NDUFC2* | 2.198 | 1.35E-63 |
| *FAM60A* | 1.742 | 1.43E-36 |
| *FGFRL1* | 1.24 | 2.60E-15 |
| *SYNDIG1* | 2.528 | 9.44E-29 |
| *TMEM45B* | 2.149 | 4.10E-41 |
| *CD24* | 8.032 | 2.42E-158 |
| *ZDHHC9* | 1.398 | 4.93E-31 |
| *AUNIP* | 1.824 | 2.97E-66 |
| *DNPH1* | 1.358 | 4.54E-31 |
| *CXCL9* | 2.335 | 1.32E-20 |
| *SIX4* | 1.418 | 4.16E-36 |
| *BCL2L1* | 1.916 | 5.94E-64 |
| *FGF18* | 3.729 | 1.18E-62 |
| *SLC45A4* | 2.005 | 3.56E-40 |
| *SLFN13* | 1.048 | 4.26E-15 |
| *BUB1B* | 2.462 | 7.33E-80 |
| *HJURP* | 3.179 | 8.55E-95 |
| *EIF2AK1* | 1.42 | 3.30E-39 |
| *POU2F3* | 2.943 | 2.99E-48 |
| *DTL* | 2.34 | 2.19E-72 |
| *RSAD2* | 1.364 | 1.21E-17 |
| *C6ORF132* | 4.65 | 1.29E-137 |
| *SMPDL3B* | 4.767 | 1.62E-145 |
| *LY6E* | 2.585 | 1.04E-49 |
| *LAPTM5* | 2.704 | 1.04E-34 |
| *SCNN1A* | 5.047 | 1.81E-117 |
| *ARF3* | 1.513 | 1.02E-38 |
| *CDCP1* | 3.283 | 4.33E-83 |
| *CD164L2* | 1.223 | 2.20E-23 |
| *COL10A1* | 2.111 | 1.67E-15 |
| *F11R* | 1.967 | 1.06E-41 |
| *GMPS* | 1.438 | 8.10E-42 |
| *GPT2* | 2.267 | 6.85E-50 |
| *TTC9* | 2.133 | 2.89E-46 |
| *NCAPG* | 2.526 | 4.04E-79 |
| *IL2RB* | 1.522 | 2.94E-21 |
| *FXYD3* | 5.415 | 1.08E-82 |
| *TUBB4A* | 2.061 | 7.02E-14 |
| *N4BP3* | 1.071 | 8.12E-23 |
| *CTSC* | 1.864 | 6.70E-38 |
